# Natural antisense transcripts as drug targets

**DOI:** 10.3389/fmolb.2022.978375

**Published:** 2022-09-27

**Authors:** Olga Khorkova, Jack Stahl, Aswathy Joji, Claude-Henry Volmar, Zane Zeier, Claes Wahlestedt

**Affiliations:** ^1^ Center for Therapeutic Innovation and Department of Psychiatry and Behavioral Sciences, University of Miami, Miami, FL, United States; ^2^ Department of Chemistry, University of Miami, Miami, FL, United States

**Keywords:** natural antisense transcript (NAT), long nocoding RNA, anisense oligonucleotides, posttranscriptional regulation, nucleic acid based therapeutics

## Abstract

The recent discovery of vast non-coding RNA-based regulatory networks that can be easily modulated by nucleic acid-based drugs has opened numerous new therapeutic possibilities. Long non-coding RNA, and natural antisense transcripts (NATs) in particular, play a significant role in networks that involve a wide variety of disease-relevant biological mechanisms such as transcription, splicing, translation, mRNA degradation and others. Currently, significant efforts are dedicated to harnessing these newly emerging NAT-mediated biological mechanisms for therapeutic purposes. This review will highlight the recent clinical and pre-clinical developments in this field and survey the advances in nucleic acid-based drug technologies that make these developments possible.

## 1 Introduction

Historically, therapeutic development efforts have focused on finding protein-targeting small molecules, predominantly with inhibitory or antagonistic effects. In such cases, the target proteins usually belong to the receptor, channel or enzyme functional classes (Hopkins and Groom, 2002). However, these requirements severely restrict the range of usable druggable disease targets, limiting it to only a small subset of protein-coding genes (Bull and Doig, 2015).

Fortuitously, the recent discovery of vast non-coding RNA-based regulatory networks that can be easily modulated by nucleic acid-based therapeutics (NBTs) has opened numerous new therapeutic possibilities. Long non-coding RNA (lncRNA) class, and its natural antisense transcript subclass (NATs) in particular, play significant roles in these networks (Wahlestedt 2013) and regulate gene expression through a wide variety of biological mechanisms, as described in this review.

**FIGURE 1:**
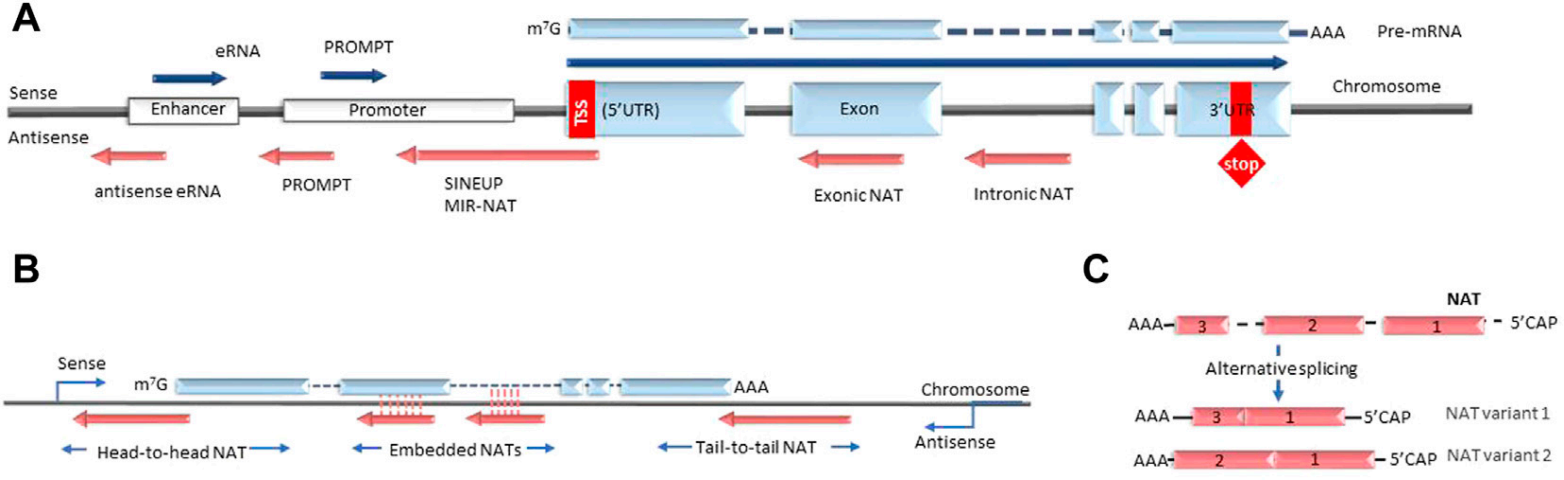
Types of antisense transcripts. **(A)** Enhancer RNAs and PROMPTs are transcribed bidirectionally from enhancer and promoter regions respectively. Intronic and exonic NATs are transcribed from the DNA strand opposite to the protein-coding gene under the control of different promoters (bidirectional, independent, latent etc.). SINEUPS are encoded antisense to the 5′ end of the mRNA and have embedded transposable elements at their 3′ends. MIR-NATs are characterized by effector domains with retrotransposon-derived repeats, such as mammalian-wide interspersed repeats (MIR). **(B)** NATs are described as head-to-head, embedded and tail-to-tail depending on their position relative to the sense transcript. **(C)** Some NATs are modified with 5′-caps and polyA tails and undergo alternative splicing. NAT, natural antisense transcript; eRNA, enhancer RNA; PROMPT, promoter upstream transcript; UTR, untranslated region; SINEUP, inverted SINEB2 sequence-mediated upregulating molecule.

lncRNA are defined as non-coding transcripts longer than 200 nucleotides. The NAT subclass of lncRNA comprises non-coding RNA that are transcribed from the antisense strand of the coding gene loci. In the current nomenclature NATs are distinct from the long intergenic non-coding RNAs (lincRNAs) that are defined as long transcripts expressed from intergenic regions. The most commonly proposed classification of NATs is based on their genomic position relative to their partner sense gene. In this case the groups usually include transcripts that are expressed from the DNA strand opposite to: 1) the coding gene’s promoter region (e.g. promoter upstream transcripts or PROMPTs); 2) coding gene introns or exons (termed overlapping or intronic/exonic NATs); 3) enhancers (e.g. eRNAs) (Figure 1) (Agostini et al., 2021). However, the utility of this grouping as a classification is limited, as many known NATs overlap several or all of these regions. Furthermore, this grouping does not fully reflect the biological functions of NATs, which arguably would be very relevant for understanding NAT diversity. However, as the lncRNA field is still in its initial exponential growth stages, the full spectrum of NAT types and NAT-dependent mechanisms is still not well studied and a comprehensive classification of NATs cannot be achieved at this time. As a result, NAT nomenclature is also not yet established and multiple synonyms are used to describe similar entities, for example “NATs”, “anti-sense lncRNA” or “AS-lncRNA” are used interchangeably. Another possible case where current nomenclature creates distinction without difference are NATs and long promoter and enhancer-generated ncRNA, that in the future could be understood as parts of the same biological mechanism discovered at different times and in different contexts. Overall, as the current understanding of lncRNA biology is uncertain we organize the review around existing definitions.

Due to their chemical composition and 3D shape, NATs can efficiently scaffold complexes that contain both proteins and nucleic acids. Furthermore, due to their complementarity to DNA and RNA sequences, NATs can guide locus-specific or mRNA-specific targeting of regulatory protein complexes with genome-wide functions. As described in this review, NAT/protein complexes are known to regulate high level chromosome structures, epigenetic state of specific gene loci, transcription, translation, trafficking, subcellular localization and post-transcriptional modification of specific genes and mRNAs.

As a result of recent successes in the development of diverse NBT modalities, including small interfering RNAs (siRNAs), antisense oligonucleotides (ASOs), therapeutic mRNAs, viral delivery vectors and others, NAT-mediated biological mechanisms are now accessible for precise gene-specific therapeutic modulation (Crooke et al., 2021).

This review will highlight the recent clinical and pre-clinical developments in the field of RNA-targeted therapeutics, survey advances in NBT technology and the increased understanding of nucleic acid-based mechanisms of action that make these developments possible.

## 2 Types of NBTs

Extensive efforts dedicated to the development of NBTs can be explained, in part, by their ability to target multiple novel therapeutic targets from the wide ncRNA-based regulatory networks, rational design, simplified development cycle and alignment with the goal of achieving personalized medicine. The ability to precisely tailor NBTs to a unique disease or even a single patient can increase the efficacy of treatment and minimize off-target effects (Wahlestedt 2013; Crooke et al., 2021).

The current range of NBTs includes multiple modalities, such as short (20–30 bases) single-stranded DNA (ssDNA) or double-stranded RNA (dsRNA) oligonucleotides, full length therapeutic mRNA and plasmid or viral-based vectors expressing single or multiple NBTs. In the cell, ssDNA oligonucleotides, termed ASOs, form a duplex with target RNA and induce its degradation by RNAse H. dsRNA oligonucleotides, termed small interfering RNAs or siRNAs, direct the target RNA to the RNA interference pathway, also leading to its degradation. Alternatively, these oligonucleotides can pair with and block protein- or RNA/DNA-binding sites on their target RNAs or, conversely, directly block the RNA-binding pocket on RNA-binding proteins. Inhibition of RNA-protein, RNA-DNA and RNA-RNA binding can also be achieved by oligonucleotide binding to any site on the nucleic acid or protein that would induce significant conformational change in these molecules. In all of these cases, oligonucleotides can be rationally designed to interact with high specificity with their target through base-pairing, with minimal off-target interactions (Khorkova and Wahlestedt 2017).

The development of NBTs was largely fueled by successes in chemical modifications of nucleic acids that increased nuclease resistance of DNA and RNA oligonucleotides. Phosphorothioate (PS) bonds and sugar moiety modifications, such as 2′O-methyl (2OMe), 2′-O-methoxyethyl (2MOE), fluoro (F), locked nucleic acids (LNAs), as well as phosphorodiamidate backbones and phosphorodiamidate morpholino oligomers (PMO), have already been used in approved drugs (Table 1; reviewed in Roberts et al., 2020). These chemically modified nucleic acids have extended tissue half-lives, especially in cerebrospinal fluid (CSF), which has low nuclease content, and are readily taken up by cells. However, they do not penetrate intestinal lining or blood-brain barrier (BBB) to a significant extent (Khorkova and Wahlestedt 2017). Currently, the solution to this problem is intravenous (IV), subcutaneous (SC), intracerebral (IC), intracerebroventricular (ICV), or intrathecal (IT) route of administration. Notably, these methods are invasive and create a significant burden for patients.

**TABLE 1:** Nucleic acid-based therapeutics and RNA-targeting small molecules^a^.

Drug name	Type	Target	Indication	Date	Company	Delivery Route
Fomivirsen (Vitravene)	RNAse H, PS ASO	UL123 gene of cytomegalovirus	Cytomegalovirus retinitis	1998	Ionis, Novartis, Abbot	Intravitreal injection
Pegaptanib (Macugen)	Aptamer, Pegylated PD, 2MOE, 2F oligo	VEGF antagonist	Age-related macular degeneration	2004	NeXstar, Gilead, OSI	Intravitreal injection
Mipomersen (Kynamro)	RNAse H, PS 2MOE ASO	ApoB100	Homozygous familial hypercholesterolemia	2013	Ionis, Kastle	SC
Eteplirsen (ExonDys51)	Exon skipping, morpholino ASO	Dystrophin (DMD)	Exon 51-related Duchenne muscular dystrophy	2016	Sarepta	IV infusion
Nusinersen (Spinraza)	Exon skipping, PS 2MOE ASO	SMN2	Spinal muscular atrophy	2016	Ionis, Biogen	IT
Inotersen (Tegsedi)	RNAse H, PS 2MOE ASO gapmer	TTR	Hereditary transthyretin-mediated amyloidosis	2018	Ionis, Akcea	SC
Patisiran (Onpattro)	siRNA in lipid nanoparticles	TTR	Hereditary transthyretin-mediated amyloidosis	2018	Alnylam	IV
Golodirsen (Vyondys 53)	Exon skipping, morpholino ASO	Dystrophin (DMD)	Exon 53-related Duchenne muscular dystrophy	2019	Sarepta	IV infusion
Milasen	Splice switching, PS 2MOE ASO	MFSD8	Batten disease	2019	Boston Children’s Hospital	IT
Givosiran (Givlaari)	siRNA, GalNac-conjugated	ALAS1	Acute hepatic porphyria	2019	Alnylam	SC
Viltolarsen (Viltepso)	Exon skipping, morpholino ASO	Dystrophin (DMD)	Exon 53-related Duchenne muscular dystrophy	2020	Nippon Shinyaku Pharma	IV
Volanesorsen (Waylivra)	RNAse H, PS 2MOE ASO	ApoCIII	Familial chylomicronaemia	2020^b^	Ionis, Akcea	SC
Lumasiran (Oxlumo)	RNAi, Enhanced Stabilization Chemistry-GalNAc	Glycolate oxidase (HAO1)	Primary hyperoxaluria type 1	2020	Alnylam	SC
Casimersen (SRP-4045, AMONDYS 45)	Exon skipping, PMO ASO	Dystrophin (DMD)	Exon 45-related Duchenne muscular dystrophy	2021	Sarepta	IV
Inclisiran (Leqvio)	siRNA, Enhanced Stabilization Chemistry-GalNAc	PCSK9	Familial hypercholsterolemia	2020^b^, 2021	Alnylam, The Medicines Company, Novartis	SC
Risdiplam (EVRYSDI®)	Splice-modulating small molecule	SMN2 pre-mRNA	Spinal muscular atrophy	2020	Roche/PTC Therapeutics	Oral
Ataluren (Translarna)	Small molecule	PTC read-through	Duchenne muscular dystrophy	2014^b^	PTC Therapeutics	Oral

a Approved by FDA, as of November 2021.

b Approved by EMA, only.

2F, 2-fluoro; 2MOE; 2′-O-(2-methoxyethyl); ALAS1, delta-aminolevulinate synthase 1; ASO, antisense oligonucleotide; EMA, the European Medicines Agency; FDA, the United States Food and Drug Administration; GalNac, N-acetylgalactosamine; IT, intrathecal; IV, intravenous; MFSD8 - major facilitator superfamily domain-containing protein 8; PCSK9, proprotein convertase, subtilisin/kexin-type, 9; PD, phosphodiester; PMO, phosphorodiamidate morpholino oligomers; PS, phosphorothioate; PTC, premature termination codon; RNAi–RNA, interference; SC, subcutaneous; siRNA, small interfering RNA; SMN2, survival of motor neuron 2; TTR, transthyretin.

Further development of chemical modifications that permit administration of NBTs via alternative delivery routes is now underway. In parallel, advanced clinical techniques with less invasive administration routes, including inhalation, oral, or minimally invasive intranasal depot (MIND), are helping to expand the use of NBTs in the clinic (Gennemark et al., 2021; Padmakumar et al., 2021). Chemical modifications are also being developed to ensure targeted delivery of NBTs to disease-relevant tissues or cell types to reduce potential side effects and improve efficacy, such as N-acetylgalactosamine (GalNAc) conjugation to target the liver or 2′-O-hexadecyl (C16) for central nervous system (CNS) targeting (Brown et al., 2022).

Furthermore, dsRNA-based NBTs, such as siRNA or small activating RNA (saRNA), and therapeutic mRNAs require a lipid-based carrier for nuclease protection, immunogenic suppression and efficient entry into cells. Currently these areas are undergoing intense development (Roberts et al., 2020).

Technologies developed in the oligonucleotide field were utilized and inspired further development of therapeutic mRNAs. Additionally, codon optimization strategies, ‘non-natural’ caps and chemical modifications of nucleotides are being utilized to increase protein expression from therapeutic mRNAs. One of the significant successes in the therapeutic mRNA field is the RNA-based COVID19 vaccines (Barbier et al., 2022).

Another type of NBT, viral-based vectors, initially used in gene therapy, is now undergoing rapid development, with several approved drugs and many ongoing clinical trials (Table 2). Gene therapy advances have generated increased interest in developing vectorized ASOs and siRNAs, primarily using adeno-associated virus (AAV) vectors (Chen W. et al., 2020). Advantages of vectorized NBTs include the possibility of infrequent dosing, systemic administration for CNS targets, directing NBTs to particular cell types, modulation of expression level and timing through the use of regulatory sequences and simplified delivery of combinatorial treatments. Vectors can also simplify expression and delivery of DNA and RNA editing constructs, such as CRISPR-CAS9, ADARs or TALENs, which may contain multiple factors (Chen W. et al., 2020).

**TABLE 2:** Gene therapy treatments approved by FDA and/or EMA^a^.

Drug name	Vector	Transgene/target	Indication	Date	Company/comments	Delivery Route
Alipogene tiparvovec (Glybera)	AAV1	Lipoprotein lipase	Lipoprotein lipase deficiency	2012^b^	AMT, UniQure; discontinued in 2017 for cost	Intramuscular injection
Talimogene laherparepvec (Imlygic/T-Vec/Oncovex)	Herpes simplex virus 1	Immunotherapy, tumor cell lysis	Melanoma	2015	BioVex, Amgen	Injection into lesions
Strimvelis	Autologous CD34^+^ stem cells/gamma-retrovirus	Adenosine deaminase (ADA)	Adenosine deaminase-severe combined immunodeficiency	2016^b^	Orchard; on hold due to mutagenesis concerns	IV infusion
Voretigene neparvovec (Luxturna)	AAV2 vector	RPE65	Leber congenital amaurosis	2017	Spark Therapeutics, Children’s Hospital of Philadelphia	Subretinal injection
Axicabtagene ciloleucel (Yescarta)	CAR-T therapy, ϒ-retroviral vector	CD-19	Diffuse large B-cell lymphoma; primary mediastinal large B-cell lymphoma	2017	Kite	IV infusion
Tisagenlecleucel (Kymriah)	CAR-T therapy, lentiviral vector	CD-19	B-cell acute lymphoblastic leukaemia, diffuse large B-cell lymphoma	2017	Novartis	IV infusion
Onasemnogene abeparvovec (Zolgensma)	AAV9	SMN1	Spinal muscular atrophy	2019	Novartis, AveXis	IV infusion
Betibeglogene autotemcel (Zynteglo)	Lentiglobin BB305 (lentiviral vector)	Beta-globin	Beta thalassaemia	2019^b^	Bluebird bio	IV infusion
Atidarsagene autotemcel (Libmeldy)	Autologous CD34^+^ stem cells/lentivirus	ARSA	Metachromatic leukodystrophy	2020^b^	Orchard	IV infusion
Brexucabtagene autoleucel (Tecartus)	CAR-T therapy, replication-incompetent retroviral vector	CD19	Mantle cell lymphoma	2020	Gilead/Kite	IV infusion
Tisagenlecleucel (Kymriah)	CAR-T therapy, lentiviral vector	CD19	B-cell acute lymphoblastic leukaemia, diffuse large B-cell lymphoma	2018	Novartis	IV infusion
Elivaldogene autotemcel (Skysona)	Autologous CD34^+^ stem cells/lentiviral vector	ALDP	Adrenoleukodystrophy	2021^b^	bluebird bio	IV infusion
Idecabtagene vicleucel (Abecma)	CAR-T-cell therapy, lentiviral vector	B-cell maturation antigen	Multiple myeloma	2021	Celgene/BMS	IV infusion
Lisocabtagene maraleucel (Breyanzi)	CAR-T therapy, replication-incompetent, self-inactivating lentiviral vector	CD19	Large B-cell lymphoma	2021	Celgene/BMS	IV infusion
Lenadogene neparvovec (Lumevoq, GS010)	Gene therapy, rAAV2/2-ND4	ND4	Leber’s hereditary optic neuropathy	Postponed to 2022	GenSight Biologics	Intravitreal injection

a Approved by FDA, as of November 2021.

b Approved by EMA, only.

AAV, adeno-associated virus; ALDP - ATP-binding cassette, subfamily D, member 1 (ABCD1); ARSA, arylsulfatase A; BMS, Bristol-Myers Squibb; CAR-T, chimeric antigen receptor T cell; CD19—CD19 antigen; EMA, the European Medicines Agency; FDA, the United States Food and Drug Administration; IV, intravenous; ND4 - complex I, subunit ND4 (MTND4); rAAV, recombinant adeno-associated virus; SMN1 - survival of motor neuron 1.

For each therapeutic goal, the modulation of relevant RNA targets may be achieved using multiple therapeutic targets and NBT modalities. For example, several different therapeutic modalities are currently being pursued in spinal muscular atrophy, including gene therapy, therapeutic mRNA delivery, small molecule modulators of splicing and ASOs targeting different biological mechanisms (Table 1, 2; Khorkova et al., 2021).

As more data about NATs involvement in regulation of well-studied and novel biological mechanisms becomes available, the applicability and therapeutic value of NAT-targeting NBTs steadily increases, further fueling the development of the NBT field.

## 3 Biological processes that can be modulated by NAT-targeted therapeutics

After the initial survey of cellular RNA conducted in the early 2000s revolutionized biology by revealing the presence of multitudes of ncRNA transcripts, extensive work has been directed at establishing their biological functions (Wahlestedt 2013; Andergassen and Rinn 2022). This work led to the emerging concept of the cell as an “RNA machine”, as it is becoming clear that ncRNA provide the scaffold and ‘gears’ and regulate the majority of biological processes as described in this section (Rinn and Chang 2020). We also review several clinically advanced and discovery-stage examples of NAT-targeting NBTs that modulate these mechanisms.

### 3.1 Regulation of transcription

lncRNA and NATs in particular are involved in the intricate regulatory networks that modulate gene expression at the level of transcription. Notably, this regulation substantially influences post-transcriptional processes as it can determine sequence and chemical modification of mRNA and its trafficking, subcellular localization and translation efficiency.

As described below, the main role of NATs in transcription complexes frequently lies in guiding their assembly at specific gene loci. Focusing on the RNA components of the transcriptional machinery facilitates development of highly gene-specific NBTs. Targeting the ‘general purpose’ protein components of transcriptional complexes on the contrary can result in multiple off-target, pan-genomic effects.

**FIGURE 2:**
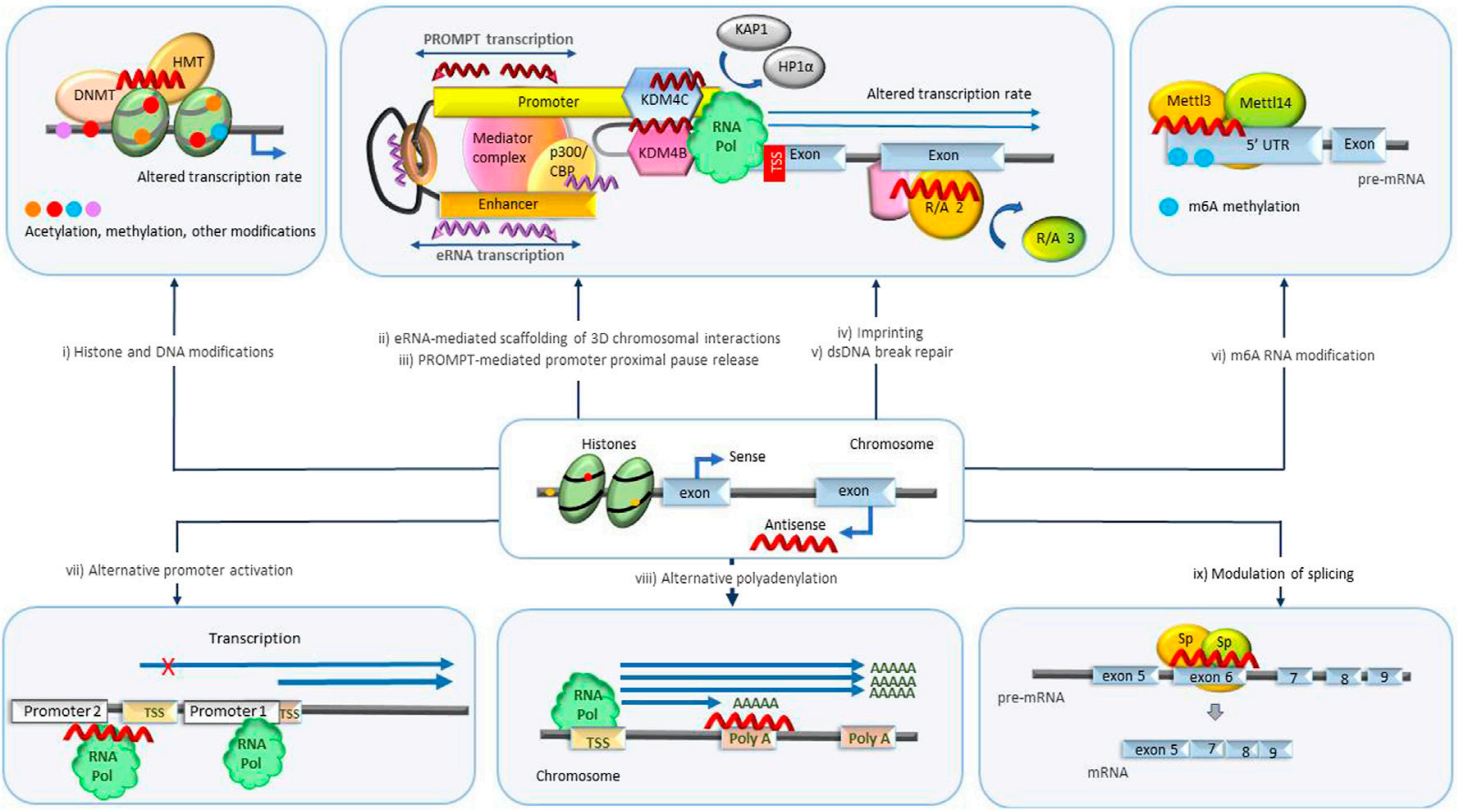
Transcriptional level biological processes regulated by NATs. (i) Histone and genomic DNA modification. NATs bound to specific loci in DNA scaffold epigenetic regulatory enzymes such as DNA methyltransferases (DNMT) and histone modifiers that mediate acetylation and methylation and initiate imprinting and changes in transcription rate. (ii) eRNA scaffolding 3D chromosomal interactions. eRNAs maintain the chromatin active state and mediate the enhancer-promoter looping by serving as decoys or otherwise modulating the functions of participating protein complexes such as BRD4, CBP, NELF, cohesin, Mediator complex and others leading to changes in transcription rate and mRNA abundance. (iii) PROMPT-mediated promoter-proximal pause release. PROMPTS initiate histone modifications by recruiting various epigenetic modifiers (KDM4B, KDM4C) triggering destabilization of protein complexes (HP1α, KAP1) involved in promoter-proximal Pol II pausing, resulting in release of Pol II and increased transcription. (iv) Imprinting. NATs scaffold/decoy/block different repressor and activator proteins catalyzing transcription repression of specific parental alleles. (v) DNA damage repair. NATs scaffold/decoy proteins involved in double-stranded DNA break repair. (vi) m6A RNA modification. NATs mediate m6A modification of specific RNA molecules by recruitment of regulators or enzymes associated with methyltransferase complexes (Mettl3/Mettl14) or m6A demethylases (ALKBH5). m6A modification alters availability and processing of RNAs. (vii) Alternative promoter activation. NATs alter the transcription rate of specific isoforms by antagonizing or activating alternative promoters. (viii) Alternative polyadenylation. NATs repress/activate polyA signals prompting transcription of alternative isoforms. ix) Modulation of splicing. NATs modulate the target binding and activity of splicing promoting/inhibiting factors (Sp) facilitating production of specific isoforms. DNMT, DNA methyl transferase; HMT, histone methyl transferase; eRNA, enhancer RNA; CBP/p300, CREB binding protein; PROMPT, promoter upstream transcripts; KDM4B/4C -lysine specific demethylase 4B/4C; UTR, untranslated region; m6A, N6-methyladenylation; TSS, transcription start site.

Regulation of transcription is highly complex and can occur at different levels (Figure 2). Besides regulation at the level of transcription initiation and elongation, specific protein levels in the cell are also regulated through co-transcriptional chemical modification of pre-mRNAs (Neil et al., 2022). These modifications include 5′ end capping, as well as intron splicing and ligation of protein-coding exons. The splicing process is regulated to include different alternative sets of exons, resulting in multiple alternative transcripts (isoforms). At transcriptional termination, the 3′ end of the nascent RNA undergoes cleavage and polyadenylation. Enzyme complexes that mediate chemical modification of individual nucleotides, resulting in their conversion into n6-adenosine (m6A), 5-methylcytosine (m5C), pseudouridine, 2-O-methyl derivatives also engage co-transcriptionally.

Transcripts that do not successfully complete these steps are detected by the mRNA surveillance machinery and targeted to RNA decay pathways, such as nonsense-mediated decay (NMD) pathway. These processing steps not only define the length and sequence of RNA transcripts, but also regulate RNA stability, localization, and translation and ultimately control transcript expression and protein levels. As described below, NATs have been reported to regulate many of these steps. In the following sections we give examples of multiple existing or proposed NBTs that can specifically modulate these NAT-mediated regulatory mechanisms.

#### 3.1.1 Imprinting

Imprinting is an epigenetic process in which expression of one of the 2 alleles of a gene is constitutively repressed. In an extreme case of imprinting, X-chromosome inactivation, the majority of genes on one copy of X-chromosome are shut down. Although imprinting mainly engages extra-long non-coding transcripts, frequently expressed over multiple gene loci and intergenic regions (Andergassen et al., 2021; Wang W. et al., 2021b), participation of NATs in imprinting has also been reported (Figures 2i, 2iv).

In some cases, targeting NATs that are known to participate in silencing of imprinted transcripts can be utilized in disease treatment. For example, in Angelman syndrome, insufficiency of ubiquitin protein ligase E3A (UBE3A) caused by mutations in maternally inherited allele can be rescued by de-repression of the imprinted paternal copy. Such de-repression could be achieved by targeting the Ube3a NAT (Ube3a-ATS) with ASOs. A single ICV injection of an anti-NAT ASO in a mouse disease model increased UBE3A protein in the brain to 74% of wild type levels. The increased UBE3A levels normalized behaviors in open field, forced swim and reversed rotarod tests and increased delta power in EEG recordings (Milazzo et al., 2021; Spencer et al., 2022). This NAT-targeting approach is being pursued by Ionis/Biogen, while an antagomir-based strategy has been proposed by Roche and collaborators (see section Regulation of miRNA activity).

#### 3.1.2 Genomic DNA methylation

Addition of a methyl group to genomic DNA, termed DNA methylation, is an enzymatic reaction underlying multiple epigenetic processes that regulate gene expression, including imprinting, epimutations, modulation of splicing and others (Figure 2i).

Several mechanisms through which NATs coordinate DNA methylation have been described. The process of antisense transcription through CpG islands could lead to dissociation of factors that maintain their methylation status. Alternatively, NATs could form RNA:DNA:DNA triplexes with genomic DNA in the promoter regions to facilitate the binding of methylation protein complexes or inhibit activity of demethylases. Triplexes can be formed by homopolypurine/homopolypyrimidine regions of duplex DNA and single-stranded RNA molecules that insert into the major groove of DNA in a parallel or antiparallel orientation. The triplex is supported by Hoogsteen hydrogen bonding between RNA and dsDNA (Li Y et al., 2016).

In a disease-relevant example, lncRNA PARTICLE (promoter of MAT2A-antisense radiation-induced circulating lncRNA) is predicted to form triplexes at >1600 genomic locations including WWOX (WW domain containing oxidoreductase) and its neighboring gene MAT2A (methionine adenosyltransferase 2A) (O'Leary et al., 2017). PARTICLE was shown to scaffold DNMT1 (DNA (cytosine-5)-methyltransferase 1) and histone tri-methyltransferases which led to reduced expression of its neighboring gene MAT2A in cis and the WWOX gene in trans (O'Leary et al., 2019). MAT2A is suggested as a therapeutic target in cancer (Li et al., 2022), while WWOX is associated with developmental and epileptic encephalopathy 28, esophageal squamous cell carcinoma and spinocerebellar ataxia, autosomal recessive 12.

In another example, activity of PEBP1P3, a NAT encoded by the opposite strand of the CD45 (protein-tyrosine phosphatase, receptor-type, C or PTPRC) locus, initiates a complex chain of events that indicates involvement of DNA methylation in alternative splicing. Knockdown of PEBP1P3 led to decreased DNA methylation of CD45 gene at intron 2 and increased trimethylation of histone H3K9 and H3K36 at the alternative exons of CD45. Knockdown of PEBP1P3 also increased the binding levels of chromatin conformation organizer CTCF (CCCTC-binding factor) at intron 2 and alternative exons of CD45. These changes resulted in altered ratio of CD45 alternative splicing isoforms that may represent a therapeutic target in autoimmune or immune deficiency diseases (Su et al., 2021).

#### 3.1.3 Epimutations

Recent studies have elucidated cases where changes in DNA methylation patterns at protein-coding genes mimic effects of disease-causing mutations by inducing haploinsufficiency. These cases are referred to as epimutations and can be separated into primary, induced by environment, and secondary, due to DNA mutations affecting components of epigenetic machinery that includes NATs.

An informative example of a NAT-mediated secondary epimutation is a case triggered by the *de novo* appearance of a novel NAT due to a splicing mutation in the peroxiredoxin (PRDX1) gene. This mutation leads to activation of several cryptic splice sites and production of a read-through novel antisense transcript encompassing the promoter of the TESK2 (testis-specific protein kinase 2) gene and the bidirectional promoter of 2 genes, MMACHC (metabolism of cobalamin associated c) and CCDC163P (coiled-coil domain containing 163). The appearance of this new antisense transcript changes DNA methylation profiles in the CpG islands of the promoters, initiated by deposition of SETD2-generated H3K36me3 marks that facilitate binding of the heterochromatin protein 1 (CBX5) and recruitment of DNMT3B1 or DNMT2B. DNMT3B1/2B mediate methylation of CpG islands and silencing of MMACHC, CCDC163P and TESK2 genes. Phenotypically, it leads to epi-cblC, an inherited disorder of intracellular vitamin B12 metabolism with hematological, neurological and cardiometabolic outcomes. Silencing the PRDX1 gene partially reduced hypermethylation at affected promoters (Oussalah et al., 2022). However, modulation of PRDX1 splicing using NBTs may be a more viable therapeutic approach as PRDX1 is an essential gene involved in mitigation of oxidative stress and tumor suppression.

A similar mechanism is involved in alpha-thalassemia type α-ZF, where a deletion mutation removes HBA1 (hemoglobin-alpha locus 1) and HBQ1 (hemoglobin-theta-1 locus) genes, initiating readthrough transcription of the antisense LUC7L (LUC7-like) gene over the HBA2 (hemoglobin-alpha locus 2) gene locus. This leads to hypermethylation of HBA2 CpG islands and silencing of HBA2 expression (Guéant et al., 2022).

Another case is described in a subset of Lynch syndrome patients where deletion of the upstream EPCAM (epithelial cellular adhesion molecule) gene leads to expression of EPCAM/MSH2 (MutS homolog 2) fusion transcripts, MSH2 promoter methylation and inhibition of MSH2 expression (Cini et al., 2019). Modulation of splicing of the readthrough NATs in these cases could be achieved using NBTs.

The mechanisms that mediate communication between successive cellular generations could be viewed as a unique case of primary epimutation. nAS25, a NAT encoded in exon 25 of the notch-1 locus, transmits information about cell cycle perturbations from mother to daughter cells and confers cell cycle adjustments in progeny. nAS25, transcribed from a bi-directional E2F1-dependent promoter in the mother cell, is accumulated over the G1 phase of cell cycle thus providing a measure of G1 length. nAS25 is transmitted from mother to daughter cells, where it binds and stabilizes notch-1 sense transcripts in G0 phase to the level defined by nAS25 abundance. Resulting modulation of notch-1 signaling reprograms G1 phase in daughter cells to compensate for perturbations that occurred in the mother cell. NBT-mediated modulation of nAS25 would permit fine control of cell cycle duration (Vujovic et al., 2021).

#### 3.1.4 Histone modifications

NATs are also known to scaffold epigenetic histone modifier complexes that regulate the expression of their sense partner in *cis*, or of a group of related genes in *trans* (Figure 2i). For example, upregulation of brain derived neurotrophic factor antisense (BDNF-AS) inhibits BDNF expression and increases the recruitment of the histone methyltransferase EZH2 (enhancer of zeste 2 polycomb repressive complex 2 subunit), resulting in the deposition of repressive H3K27me3 marks at regulatory regions of the BDNF gene. ASOs, termed “AntagoNATs,” targeted to BDNF-AS induced gene-specific upregulation of BDNF *in vitro* and *in vivo* (Modarresi et al., 2012)*.* Furthermore, treatment with the BDNF AntagoNAT delivered by MIND technique induced widely distributed upregulation of BDNF protein in rat brains. Compared to intranasal delivery through inhalation, MIND allows precise dosing and represents a patient-friendly brain delivery route for NBTs (Padmakumar et al., 2021). BDNF upregulation can be beneficial in many neurodegenerative diseases including Parkinson’s and Alzheimer’s disease (Joshi and Salton, 2022).

Triplex formation can also be involved in histone modification. For example, an antisense transcript DLX6-AS1 formed a triplex structure at the promoter region of DLX6 (distal-less homeobox 6) gene, facilitating the recruitment of histone acetyltransferase p300 and E2F1 and thereby promoting transcription of DLX6 in endometrial cancer. Additionally, DLX6-AS1 could enhance cell proliferation and cancer development by sponging multiple miRNAs, including miR-26a (leading to up-regulation of PTEN), miR-203a (targeting MMP2), miR-197-5p (to relieve E2F1) and miR-199a. Silencing DLX6-AS1, on the contrary, inhibited tumor growth (Zhao and Xu, 2020).

Similar mechanisms are likely involved in SCN1ANAT-mediated downregulation of SCN1A expression. Treatment with AntagoNATs induced specific upregulation of SCN1A both *in vitro* and *in vivo* and significantly improved disease phenotype in a mouse model of Dravet syndrome (Hsiao et al., 2016). This approach in Dravet syndrome treatment is currently being pursued by CuRNA/CAMP4 Therapeutics.

In another disease-relevant example, the antisense transcript FOXP4-AS1 (forkhead box P4 antisense 1) suppressed ZC3H12D (zinc finger ccch domain-containing protein 12D) expression by facilitating EZH2 recruitment and subsequent deposition of repressive H3K27me3 marks, thus promoting the progression of hepatocellular carcinoma (HCC) (Ye et al., 2021). Knockdown of FOXP4-AS1 may, therefore, be therapeutically beneficial in HCC.

Another antisense transcript, FAM83C-AS1 (family with sequence similarity 83 member C antisense 1) inhibits expression of SEMA3F (semaphorin 3F) through H3K27me3 methylation at the SEMA3F promoter. Stabilization of EZH2 is achieved by FAM83C-AS1-mediated recruitment of deubiquitinase ZRANB1 (zinc finger RANBP2-type containing 1). Inactivation of SEMA3F is associated with the development of colorectal cancer (Xue et al., 2020).

A more complex chain of events was triggered by the antisense transcript CDKN2B-AS1 (cyclin-dependent kinase inhibitor 2B antisense). CDKN2B-AS1 recruited the CREBBP (CREB-binding protein) and SMYD3 (SET and MYND domain-containing 3) epigenetic-modifying complex to the promoter of NUF2 (Ndc80 kinetochore complex component) to facilitate H3K27ac and H3K4me3 deposition, thereby epigenetically activating NUF2 transcription. At the same time, CDKN2B-AS1 was stabilized through its interaction with IGF2BP3 (insulin-like growth factor 2 mRNA-binding protein 3). IGF2BP3 is upregulated in renal clear cell carcinoma together with CDKN2B-AS1 and NUF2. CDKN2B-AS1 knockdown suppressed cell proliferation and invasion *in vitro* and *in vivo* (Xie X. et al., 2021).

#### 3.1.5 3D chromosomal interactions

Gene expression regulation involves the formation of three dimensional (3D) chromosomal structures that bring genomic regulatory elements, such as promoters or enhancers, into spatial proximity to each other (Figure 2). These interactions are frequently mediated by CTCF, Yin Yang 1 (YY1) and the Mediator and cohesin complexes and scaffolded and regulated by different lncRNA subtypes, including NATs (Rowley and Corces 2018).

One such NAT, STX18-AS1 (syntaxin 18 antisense 1), antagonized CTCF-mediated repressive 3D promoter interactions at the MSX1 (Msh Homeobox 1) gene, thereby augmenting MSX1 gene expression. MSX1 is a transcription factor essential for embryogenesis and cancer development (Liu et al., 2021).

In another disease-relevant example, transcription of an ncRNA ThymoD (thymocyte differentiation factor) promoted demethylation at CTCF bound sites and activated cohesin-dependent looping which led to translocation of the Bcl11b (BAF chromatin remodeling complex subunit BCL11B) enhancer from the lamina to the nuclear interior. This led to the alignment of Bcl11b enhancer and promoter in a single loop and deposition of activating epigenetic marks across the loop (Isoda et al., 2017). Bcl11b is a zinc finger transcription factor that regulates hematopoietic progenitor cell development which is important in the treatment of multiple conditions.

#### 3.1.6 Modulation of splicing

Another layer of regulation that affects the exact sequence and therefore the biological activity of the resulting protein comes into play during excision of intronic sequences from pre-mRNAs. RNA splicing is currently viewed as a tightly regulated co-transcriptional process that generates sets of mRNA isoforms, frequently with different functions, from the same pre-mRNA/genomic DNA sequence (Figure 2ix).

The sequence of each isoform is determined by divergent use of the 5′ (donor) and 3′ (acceptor) splice sites in the pre-mRNA sequence. The splice site sequences are characterized by the presence of a 5′ sequence marked by the GU dinucleotide at the extreme 5′ end of the intron, followed by a downstream ‘branch point’ sequence marked by an A, a region high in pyrimidines (C and U, termed polypyrimidine tract) further downstream, and a splice acceptor site marked by an AG sequence that terminates the intron at the 3′ end. The usage of the splice sites can be regulated by lncRNAs (Morrissy et al., 2011; Romero-Barrios et al., 2018) and protein splicing factors that recognize the lncRNA or specific sequence motifs on the intronic or exonic side of the splice site. These lncRNA and protein factors are expressed in a tissue and development stage-specific manner (Pisignano and Ladomery, 2021).

Furthermore, the multi-step splicing reaction is catalyzed by a ribonucleoprotein complex, termed spliceosome, that assembles at introns, as guided by the lncRNAs and splicing factors. The core spliceosome is composed of five small nuclear ribonucleoproteins (snRNPs) that contain small nuclear RNAs (snRNAs). snRNA U1 pairs with the 5′ splice sites in pre-mRNAs. The 3′ splice site and the polypyrimidine tract are recognized by U2 auxiliary factors 1 and 2 (U2AF1 and U2AF2). The branch point sequence at the splice site is bound by U2 snRNP. As a result, the splicing process extensively relies on RNA-RNA and RNA-protein interactions and can be readily targeted by NBTs.

Indeed, multiple splice-modulating NBTs targeting pre-mRNA elements essential for splicing have been proposed (Sergeeva et al., 2022). Nusinersen (SPINRAZA®), a splice-switching ASO, was approved by the FDA in 2016 for the treatment of spinal muscular atrophy (SMA). Nusinersen inhibits skipping of exon 7 in SMN2 by binding to the intronic splicing silencer N1 (ISS-N1), a complex regulatory element located immediately downstream of exon 7. This leads to exon 7 retention and increased production of functional SMN2 protein. Other splice switching ASOs that are currently used in the clinic are listed in Table 1.

Although not yet at the clinical stage, NBTs targeting NATs that modulate RNA splicing have been considered. Knockdown of NATs frequently leads to altered expression of individual splice isoforms, which is of particular importance in cases where isoforms exhibit distinct physiological functions and/or full gene knockdown leads to serious adverse effects.

One such case is the antisense transcript ZEB2-AS1 (zinc finger E box-binding homeobox 2 antisense 1) that binds the 5′UTR of Zeb2 pre-mRNA after epithelial–mesenchymal transition in cancer. Upon binding, ZEB2-AS1 modulated the spliceosome activity, facilitating the retention of an intron containing internal ribosome entry site (IRES) in Zeb2 mRNA. The IRES promotes cap-independent translation of Zeb2 protein and down-regulates E-cadherin promoting cancer development (Mahboobeh et al., 2020).

Another example is Trdn-as, a cardiac-specific NAT of triadin gene that facilitated the recruitment of serine/arginine splicing factors and increased the levels of Trisk32, the cardiac-specific isoform of triadin. Knockout of Trdn-as in mice downregulated Trisk32, impaired Ca2^+^ handling and increased susceptibility to cardiac arrhythmias in response to catecholamine challenge (Zhao et al., 2022). Introduction of Trdn-as mimic or Trdn-as upregulation by other means can be beneficial in the treatment of cardiac arrhythmias.

Furthermore, lncRNAs, including NATs, can regulate splicing by modifying chromatin conformation and epigenetic modification of genomic DNA as described in Sections 3.1.2–3.1.5.

#### 3.1.7 Use of alternative promoters

In some cases RNA isoforms are generated through the usage of alternative promoters. Differential activation of these promoters can be regulated by NATs (Figure 2vii). RNA-seq data from 23 normal and cancerous tissues identified more than 100 NATs whose expression correlated specifically with the activity of only a subset of promoters of their sense partners (Bellido Molias et al., 2021).

For example, silencing of ENSG00000259357 and ENSG00000255031, NATs from CERS2 (ceramide synthase 2) and CHKA (choline kinase, alpha) loci, respectively, altered the promoter usage of CERS2 and CHKA. HNF4A-AS1L selectively activated the P1 promoter of HNF4A (hepatocyte nuclear factor 4-alpha), which is not associated with malignancy, while having no effect on the oncogenic P2 promoter (Bellido Molias et al., 2021).

#### 3.1.8 Polyadenylation

Another way of generating alternative isoforms is through the usage of alternative polyadenylation sites (Figure 2viii). 3′ end cleavage and polyadenylation of mRNAs is directed by the polyA signal consisting of a conserved upstream hexanucleotide element and a variable downstream GU-rich region.

The polyA signal is recognized and proteolytically processed by a large protein complex consisting of four main subcomplexes (the cleavage and polyadenylation factor (CPSF), cleavage stimulation factor (CSTF), and cleavage factors I and II (CFI and CFII)). PolyA polymerase (PAP) then adds a polyA tail to the 5′ end of the cleavage product. PABPN1 (polyA binding protein 1) binds to the expanding polyA tail and controls its length by disrupting interactions between CPSF and PAP (Neil et al., 2022).

NATs are known to be involved in the regulation of polyadenylation. One such example is AtLAS, a NAT from the synapsin II locus. AtLAS regulates alternative polyadenylation of synapsin II to increase expression of the synapsin 2b (syn2b) isoform. Syn2b binds the AMPA receptor (AMPAR) at the postsynaptic site and reduces AMPAR-mediated excitatory synaptic transmission. In mice, silencing or overexpression of AtLAS increased or decreased the social rank, respectively (Ma et al., 2020).

The RNase P-mediated endonucleolytic cleavage plays a crucial role in the 3′ end processing and thus cellular localization of mRNA. TALAM1, a widely expressed NAT at the lncRNA MALAT1 (metastasis-associated lung adenocarcinoma transcript 1) locus, promoted the 3′ end cleavage and maturation of oncogenic MALAT1 RNA. TALAM1 preferentially localized at the site of transcription and directly interacted with MALAT1. Depletion of TALAM1 impairs 3′ end cleavage and reduces the levels of MALAT1. Transcription and stability of TALAM1 is positively regulated by MALAT1, establishing a feed-forward positive regulatory loop (Zong et al., 2016).

#### 3.1.9 N6-methyladenosine (m6A) modification of RNA

N6-methyladenosine (m6A) methylation of RNA is a reversible modification that can affect alternative splicing, transport, stability and translation of mRNAs (Figure 2vi). m6A is observed in pre-mRNAs, promoter upstream transcripts (PROMPTs), NATs and enhancer RNAs (eRNAs). Introduction of m6A modification protects nascent RNAs from Integrator-mediated termination and promotes productive transcription (Xu et al., 2022).

m6A is deposited co-transcriptionally, likely by the METTL3/METTL14/WTAP m6A methyltransferase complex (MTC) within nuclear speckles. m6A can be dynamically removed by “erasers” such as FTO (FTO alpha-ketoglutarate-dependent dioxygenase) or ALKBH5 (AlkB homolog 5, RNA demethylase) in response to environmental stimuli including memory formation, cancer development and stress responses (Wang et al., 2020; Vaasjo 2022).

m6A methylation of 3′UTRs and coding regions of mRNA is epigenetically regulated by deposition of H3K36me3. One example of NAT-regulated m6A deposition on a 3′UTR is GATA3 (GATA-binding protein 3) mRNA. GATA3-AS NAT binds to GATA3 mRNA and scaffolds m6A deposition on its 3′ UTR mediated by VIRMA (vir-like m6A methyltransferase-associated protein or KIAA1429). This leads to separation of the RNA-stabilizing protein HuR, degradation of GATA3 pre-mRNA, tumor growth and metastasis (Lan et al., 2019).

m6A methylation of 5′UTRs is believed to be driven by NATs that bind directly to the 5′UTR and/or by recruitment of methyltransferase complexes (possibly Mettl14/Mettl3). miRNAs were also shown to mediate binding of Mettl3 to target sites on mRNAs. m6A modification of 5′UTRs is thought to be involved in cap-independent translation and thus facilitates the cellular stress response (Vaasjo 2022).

In a disease-relevant example, UBA6-AS1 (ubiquitin like modifier activating enzyme 6 antisense RNA 1) directly associates with UBA6 mRNA and increases its m6A methylation by recruiting RBM15 (RNA binding motif protein 15), a key regulator of m6A deposition. IGF2BP1 (insulin like growth factor 2 mRNA binding protein 1) was identified as the m6A reader protein for this modification which enhanced UBA6 mRNA stability. Increased expression of UBA6 and UBA6-AS1 suppresses proliferation, migration and invasion of ovarian cancer cells (Wang and Chen 2022).

Removal of m6A modification can also be controlled by NATs. For example, FOXM1-AS increases the interaction of FOXM1 (forkhead box M1) and m6A demethylase ALKBH5. Demethylation of FOXM1 increased both FOXM1 expression and tumor growth. Depleting ALKBH5 or FOXM1-AS disrupted glioblastoma stem-like cell tumorigenesis (Zhang et al., 2017).

The m6A modification is also used to modulate NAT activity. For example, ALKBH5 demethylase-mediated m6A removal from KCNK15-AS1 (potassium channel, subfamily K, and member 15 antisense 1) upregulated KCNK15-AS1 levels and inhibited pancreatic cancer progression. KCNK15-AS1 bound to the 5′UTR of KCNK15 and inhibited KCNK15 translation. KCNK15-AS1 also recruited the MDM2 proto-oncogene (MDM2) to promote REST (RE1 silencing transcription factor) ubiquitination leading to upregulation of PTEN and suppression of the AKT pathway (He et al., 2021).

Introduction of m6A modification into synthetic siRNAs and other NBT subtypes can increase their potency and specificity. For instance, addition of m6A in an siRNA targeting Factor VII increased siRNA’s inhibitory activity and specificity *in vitro* (Rydzik et al., 2021).

Although yet therapeutically untapped, modulation of m6A modification using NBTs could allow context-dependent control of disease-relevant protein expression. Due to intense investigations into the role and changes in m6A patterning in brain development, neuronal activity and epithelial-mesenchymal transition, this goal may be achieved in the near future (Chen Y. et al., 2020; Vaasjo 2022).

#### 3.1.10 Promoter and enhancer RNAs

Recent technological advances in detection and manipulation of low frequency/short half-life transient transcripts have definitively demonstrated the existence and biological roles of antisense transcripts generated by promoters and enhancers (Figures 2ii, iii). Furthermore, these studies have shown that bidirectional transcription of short ncRNAs is a pervasive feature of accessible chromatin, including not only enhancers and promoters, but also other DNase hypersensitive regions that do not have canonical enhancer/promoter histone modification profiles (Young et al., 2017). Conceivably, this seemingly random transcription may originate from other, yet undiscovered, regulatory elements and/or reflect transposon-associated transcription (Lanciano and Cristofari, 2020; Nair et al., 2022).

Interestingly, both promoters and enhancers produce a similar repertoire of bidirectionally transcribed short and long non-coding transcripts that participate in control of the transcription process, which could indicate their common evolutionary origins. It is also notable that, as opposed to promoters, the long transcripts originating from enhancers are predominantly ncRNA. In fact, detailed analysis of expression profiles of 27,919 human lncRNA genes across 1,829 samples of human primary cell types and tissues has demonstrated that majority of intergenic lncRNAs are transcribed from enhancers (Hon et al., 2017).

Although the composition of the promoter and enhancer non-coding transcripts is very diverse, some of them are over 200 nucleotides long and are transcribed from the antisense strand in coding gene loci, thus qualifying as NATs and adding one more layer of NAT-mediated regulation of transcription process.

##### 3.1.10.1 Promoter RNAs

Promoter antisense RNAs (pRNAs, also abbreviated as promoter antisense RNA (PAS) or promoter upstream transcripts, or PROMPTs) are generated, with low frequency, by the same promoter region as their partner coding gene transcripts, but are transcribed in the opposite direction, a process sometimes termed “divergent transcription” (Seila et al., 2008). An asymmetrical transcription efficiency of pRNA and mRNA is achieved through formation of two separate transcription pre-initiation complexes. pRNA-specific complex assembly can be promoted by the presence of R-loops and high density of poly(A) sites in the vicinity of their transcription start site. Meanwhile, the region next to the coding counterpart transcription start site is enriched in U1 snRNP recognition sites that facilitate productive elongation by Pol II. Furthermore, pRNA transcription may be terminated early by the Integrator complex that also controls transcription termination of small nuclear RNAs (snRNAs) and enhancer-derived RNAs (eRNAs). Early termination of pRNA targets them for fast degradation mediated by the nuclear RNA exosome complex (Yang 2022). Although the sequences or pRNAs from different promoters are highly diverse, they share a similar stem-loop cluster that recruits the H3K9me3 demethylases KDM4B and KDM4C (Yang 2022).

Divergent antisense transcription at promoters can be suppressed by the ZWC complex that is composed of ZC3H4 (zinc finger CCCH domain-containing protein 4), WDR82 (WD repeat-containing protein 82) and CK2 (casein kinase 2). The ZWC complex phosphorylates SPT5, a subunit of the transcription-elongation factor DSIF, resulting in divergent transcription inhibition. Reduced ZWC activity, imposed by depletion of ZC3H4, increases divergent transcription (Park et al., 2022).

One of the recently elucidated functions of pRNA is Pol II promoter-proximal pause release. In a therapeutically relevant example, induction of pRNA transcription by activated estrogen receptor-α (ERα) at more than 800 ERα target genes led to recruitment of H3K9me3 demethylases KDM4B (lysine-specific demethylase 4B) and KDM4C (lysine demethylase 4C) mediated by compact stem-loop structures in pRNA. This resulted in significant loss of H3K9me3 and release of HP1α and KAP1 factors from target gene promoters, thereby destabilizing the 7SK snRNP (small nuclear ribonucleoprotein) complex and NELFA (negative elongation factor complex, member A) that mediate promoter-proximal Pol II pausing (Yang F. et al., 2021). Introduction of pRNA mimics or pRNA minimal constructs could induce gene-specific RNApol II pause release and activation of a target gene. This could be essential for elimination of side effects in multiple diseases treated with steroids.

As pRNAs represent the gene-specific component of the pause-release mechanism, NBTs targeted to pRNA allow for fine modulation of transcription and are likely to have fewer side effects than therapeutics targeting general purpose protein factors, thereby increasing the therapeutic potential.

##### 3.1.10.2 Enhancer RNAs

Enhancer RNAs (eRNAs) are “pervasive transcripts” generated by RNA Pol II. They are rapidly degraded by the nuclear exosome complex after 3′ endonucleolytic cleavage by the Integrator complex (Integrator) approximately 1–3 kb downstream of the transcription start site. Notably, PAF1C (polymerase-associated factor 1 complex) has a role in termination of eRNAs by facilitating recruitment of Integrator (Li W. et al., 2016; Liu et al., 2022). At the same time, enhancers can also initiate transcription of lncRNA, frequently in the antisense direction to the coding gene in the same locus.

Both long and short eRNAs play important roles in modulating transcription by regulating chromatin accessibility, histone modification and Pol II pausing through different mechanisms (Lam et al., 2014; Li W. et al., 2016; Nair et al., 2022). One of such mechanisms is eRNA-mediated scaffolding of chromatin loops that bring enhancers in proximity to their target gene loci (Melo et al., 2013; Nair et al., 2022).

Furthermore, eRNAs were shown to form local RNA-DNA duplexes (R-loops). For instance, 5′ capped antisense eRNA PEARL (Pcdh eRNA associated with R-loop formation) is transcribed from the protocadherin α (Pcdh) HS5-1 enhancer region and regulates Pcdhα gene expression by forming R-loops within the HS5-1 enhancer region. Upregulation of PEARL led to strengthened local three-dimensional chromatin organization within the Pcdh topologically associating domains (Zhou Y. et al., 2021).

In another mechanism of action, eRNAs were shown to scavenge NELF from paused RNApol II, which led to RNApol II phosphorylation by positive transcription factor b (P-TEFb) complex and subsequent activation of pause-controlled genes.

eRNAs may also increase their partner gene transcription rates by modulating histone modification. For instance, eRNAs are known to stimulate the histone acetyltransferase activity of CBP (CREB-binding protein) and coordinate assembly of transcription factors, such as YY1 and BRD4 (bromodomain-containing protein 4) at regulatory elements to modulate transcription (Gorbovytska et al., 2022). Interaction between eRNA and CBP acetyltransferase can be regulated by Ago1 that controls global H3K27ac deposition. For example, Ago1 is specifically required for expression of myogenic differentiation 1 (MyoD) and downstream myogenic gene activation (Fallatah et al., 2021).

CRED9 (CEBPA regulatory elncRNA downstream 9 kb) is an enhancer-associated long non-coding RNA (elncRNA), transcribed from an enhancer located 9 kb downstream from the transcriptional start site of CEBPA (CCAAT enhancer-binding protein alpha). CRED9 positively regulates the expression of CEBPA by maintaining H3K27ac levels at the +9 kb CEBPA enhancer. Regulation of CEBPA expression has dramatic implications for the treatment of acute myeloid leukemia and multiple other diseases (Su et al., 2022; Setten et al., 2021).

In another disease-relevant example, experience-induced eRNA ADRAM (activity-dependent lncRNA associated with memory) is expressed from an enhancer located ∼500 nucleotides upstream of the Nr4a2 (nuclear receptor subfamily 4, group A, member 2) TSS. ADRAM binds directly to Nr4a2 gene through a 25 nucleotide-long region complementary to exon III of ADRAM. The binding is associated with increased accumulation of H3K4me3 at the Nr4a2 promoter and ADRAM-mediated scaffolding of the chaperone protein 14-3-3 to the Nr4a2 promoter immediately after fear extinction training. This results in displacement of HDAC3 and HDAC4, followed by CBP binding at the Nr4a2 promoter and Nr4a2 expression activation. It has been shown that ADRAM is necessary for the formation of fear extinction memory which has therapeutic significance in anxiety disorders (Wei et al., 2022).

##### 3.1.10.3 Regulation of eRNA expression

Expression rates and activity of eRNAs are dynamically regulated by diverse mechanisms and are cell type and cell cycle-dependent. One of eRNA-targeting regulatory mechanisms is mediated by p53 tumor suppressor protein that was shown to bind multiple enhancer regions and stimulate production of eRNAs that were required for induction of a p53-dependent cell-cycle arrest (Melo et al., 2013).

The activity of eRNAs and enhancers can also be regulated by NATs that are transcribed from the same or different loci. For example, KHPS1 (sphingosine kinase 1a antisense transcript) forms a triple-helical RNA:DNA:DNA structure at the SPHK1 enhancer. The resulting triplex recruits E2F1 and p300 and activates transcription of the eRNA-Sphk1, which evicts CTCF. CTCF is an architectural protein that insulates the enhancer from the SPHK1 promoter, therefore its removal activates transcription of SPHK1 (sphingosine kinase 1). Interestingly, when triplex-forming region of KHPS1 was replaced with a triplex-forming region from the lncRNA MEG3, that normally regulates TGFBR1 (transforming growth factor-beta receptor, type I), KHPS1 associated with TGFBR1 locus thus altering the regulation of TGFBR1 expression (Blank-Giwojna et al., 2019). This mechanism can be potentially utilized in NBT design.

Furthermore, eRNA activity may be regulated by RNA editing (Mancini and Li, 2021). N6-methyladenylation (m6A)-modified eRNAs are associated with highly active enhancers that recruit the nuclear m6A reader YTHDC1. In so doing, they mediate phase-separation into liquid-like condensates that then facilitate the formation of BRD4 coactivator condensate (Lee et al., 2021).

Notably, eRNA expression patterns are known to be tissue-specific. Such local eRNAs represent potential therapeutic targets downstream of glucocorticoid receptor (GR), for example, and can be used to modulate glucocorticoid response in a cell type-specific manner to reduce the possibility of side effects (Greulich et al., 2021).

Expression of eRNA is responsive to environmental stimuli. For instance, eRNAs are preferentially expressed at inflammation-related genes in response to inflammatory stimuli (Wan et al., 2022). Global run-on sequencing (GRO-seq) has demonstrated that expression of eRNA is altered in response to early-life undernutrition (Wang Y. et al., 2021). In neurons, eRNAs are expressed at immediate early genes after stimulus. To give a specific example, BRG1 (SWI/SNF-related, matrix-associated, actin-dependent regulator of chromatin, subfamily a, member 4; SMARCA4), a core subunit of SWI/SNF-like BAF ATP-dependent chromatin remodeling complexes, is recruited to multiple enhancers in an H3K27Ac-dependent manner in response to neuronal stimulation-induced, CaMKII-mediated phosphorylation. This affects cohesin binding, enhancer-promoter looping, RNA polymerase II recruitment, and enhancer RNA expression (Kim et al., 2021).

Super enhancers are clusters of enhancers that usually regulate genes linked to cell fate specification that generate super enhancer RNAs (seRNA). Importantly, activation of seRNAs is cell and tissue-specific and may serve as a target for fine modulation of cell fate (Xiao et al., 2021).

##### 3.1.10.4 Enhancer and promoter RNA-targeting NBTs

Several methods for targeting promoter RNA or modulating eRNA expression have been proposed. For instance, a repurposed RNA-guided RNA-targeting CRISPR-Cas13 machinery was used to specifically degrade eRNAs produced by an enhancer located 30 kb upstream of colony stimulating factor 1 (CSF1) (Lewis et al., 2022).

Furthermore, saRNAs, synthetic dsRNAs containing a guide strand complementary to promoter or enhancer regions of a target gene, were shown to induce gene expression (Li et al., 2006). Several possible mechanisms underlying the activity of saRNA have been proposed. saRNA may bind nascent promoter-associated transcripts or long non-coding RNAs in sense and/or antisense orientation leading to epigenetic changes within the promoter region. Alternatively, saRNAs could potentially act as microRNA mimics, as some microRNA are reported to bind promoter regions and activate gene expression. It is also possible that saRNA block the activity of promoter and enhancer RNAs (Matsui et al., 2010; Tan et al., 2021).

The activity of saRNAs may involve their recognition by dsRNA loading factors, followed by AGO2 protein binding. The passenger strand of saRNA is then discarded, and a complex consisting of guide saRNA strand, AGO2, and heterogeneous nuclear ribonucleoproteins (hnRNPs) is formed. This complex is imported into the nucleus where it binds to chromosomal DNA and participates in the RNA-induced transcriptional activation (RITA) complex. RITA interacts with RNA polymerase II to initiate transcription, leading to target gene upregulation through reduced acetylation at histones H3K9 and H3K14, increased di/trimethylation at histone H3K4, reduced dimethylation of H3K9, increased dimethylation of H3K4 and monoubiquitination of H2B at the target gene locus (Wang J. et al., 2018).

MiNA Therapeutics’ saRNA-based NBT, MTL-CEBPA, is currently being evaluated in multiple clinical trials as a supplemental therapy to enhance the efficacy of standard-of-care cancer drugs in hepatocellular carcinoma (Hashimoto et al., 2021). MTL-CEBPA consists of amphoteric iminolipid nanoparticles called SMARTICLES and CEBPA-51, a 21-mer 2′O-Me modified dsRNA. It is delivered by intravenous infusion. MTL-CEBPA treatment resulted in tumor regression in 26.7% of HCC patients with underlying viral etiology. The mechanism of action of MTL-CEBPA is likely mediated by inactivation of immune-suppressive myeloid cells induced by upregulation of transcription factor C/EBPα (Hashimoto et al., 2021).

A second saRNA NBT, designed to upregulate STING (stimulator of interferon response cGAMP interactor 1) aims to address immune evasion and improve the effectiveness of existing cancer immunotherapies and is now at early developmental stages at MiNa.

#### 3.1.11 dsDNA break repair

NATs have also been shown to regulate DNA break repair which can have implications for protein expression (Figure 2v). In a disease-relevant example, NPPA-AS1 (natriuretic peptide A antisense RNA 1) competitively binds to the splicing factor SFPQ (splicing factor proline- and glutamine-rich) displacing NONO (non-POU domain-containing octamer-binding protein) from the SFPQ/NONO dimer that is required for double-strand DNA break repair. NPPA-AS1 deletion decreased DNA damage and activated cardiomyocyte cell cycle re-entry. Loss of NPPA-AS1 promoted cardiomyocyte proliferation and exerted a therapeutic effect against myocardial infarction in mice (Fu et al., 2022).

### 3.2 Post-transcriptional regulation

**FIGURE 3:**
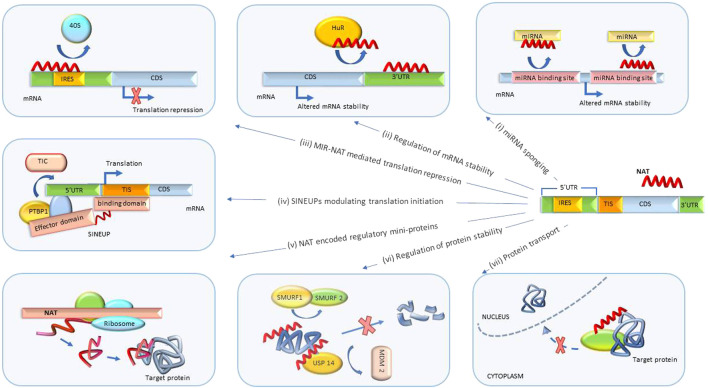
Post-transcriptional biological processes regulated by NATs. (i) miRNA sponging. NATs regulate mRNA degradation by sponging miRNA or blocking miRNA binding sites. (ii) Regulation of mRNA stability. NATs sponge proteins regulating mRNA degradation and stability. (iii) MIR-NAT-mediated translation repression. MIR-NATs overlapping 5′UTRs head-to-head compete with IRES for 40S ribosome subunit and repress translation. (iv) SINEUPs modulating translation initiation. The binding domain of SINEUP overlaps the translation initiation site and the effector domain recruits proteins (such as PTBP1) mediating assembly of translation initiation complex (TIC), resulting in translation upregulation. (v) Regulation by NAT-encoded mini-proteins. NATs can encode short peptides with downstream regulatory functions. (vi) Regulation of protein stability. NATs protect target proteins from degradation by sponging/inhibiting activity of proteins involved in ubiquitin-proteosome degradation pathway (such as SMURF1/2, MDM2). (vii) Protein translocation. NATs modulate subcellular distribution of proteins. UTR, untranslated region; CDS, coding sequence; MIR-NAT, mammalian-wide interspersed repeat natural antisense transcript; IRES, internal ribosome entry site; HuR, Hu-antigen R/ELAV-like RNA-binding protein 1; SINEUP, inverted SINEB2 sequence-mediated upregulating molecules; TIC, translation initiation complex; PTBP1, polypyrimidine tract binding protein-1; TIS, translation initiation site; USP14, ubiquitin-specific protease 14; SMURF1/2, SMAD-specific E3 ubiquitin protein ligase 1; MDM2, MDM2 proto-oncogene.

NATs are also involved in direct regulation of translation (Figure 3). Although none of the NBTs targeting these regulatory processes have advanced to clinical testing at this time, extensive research and development work is currently underway. We review several examples below.

#### 3.2.1 SINEUPs

A newly discovered class of NATs termed SINEUPs (inverted SINEB2 sequence-mediated upregulating molecules) is encoded antisense to the 5′ end of the target sense mRNA and can enhance its translation without upregulating mRNA levels (Figure 3iv). SINEUP NATs are distinguished by the presence of specific binding and effector domains. The binding domain is formed by an antisense region overlapping the start codon of target mRNAs and confers specificity to the protein coding transcript. Effector domains at the 3′ end of a SINEUP comprise embedded transposable element sequences, such as inverted short interspersed nuclear element B2 (invSINEB2), e.g. Alu or MIR, that are capable of upregulating translation by binding activating protein complexes (Podbevšek et al., 2018).

It has been shown that co-localization of a SINEUP and its mRNA target in the cytoplasm is essential for SINEUP activity. Sub-cellular distribution of SINEUPs and assembly of translational initiation complexes is mediated by RNA-binding proteins PTBP1 (polypyrimidine tract binding protein-1) and HNRNPK (heterogeneous nuclear ribonucleoprotein K). SINEUPs associate with PTBP1 and recruit ribosome subunits to target mRNAs which leads to formation of EF1A1 (eukaryotic translation elongation factor 1, alpha-1)-containing translational initiation complexes (Toki et al., 2020a).

Interestingly, endogenous SINEUP Uchl1-AS (ubiquitin carboxyterminal hydrolase L1 antisense) localizes mainly to the nucleus under normal physiological conditions, but translocates to the cytoplasm under rapamycin-induced stress, leading to increased allocation of Uchl1 mRNA to heavy polysomes (Arnoldi et al., 2022). Furthermore, the Alu monomer sequence embedded in Uchl1-AS is known to bind ILF3 (IL enhancer-binding factor 3), involved in RNA splicing and nuclear retention (Fasolo et al., 2019; Arnoldi et al., 2022). Dysregulation of UCHL1 expression is associated with spastic paraplegia 79 and susceptibility to Parkinson’s disease.

In another disease-relevant example, overexpression of the endogenous SINEUP RAB11B-AS1 (ENSG00000269386) enhanced translation of its partner sense gene RAB11B (RAS-associated protein RAB11B), with only modest upregulation of transcription. Mutations leading to RAB11B haploinsufficiency are associated with intellectual disability and microcephaly. RAB11B-AS1 is downregulated in autism spectrum disorder related to CHD8 haploinsufficiency (Zarantonello et al., 2021). Introduction of synthetic minimal RAB11B-AS1-based constructs represents a possible therapeutic target in these conditions.

Indeed, synthetic miniSINEUP molecules, which combine essential elements of binding domains complementary to a specific gene and invSINEB2 effector domains, have already been proposed (Toki et al., 2020b; Arnoldi et al., 2022). Therapeutic miniSINEUP RNA constructs are transcribed *in vitro*, purified and transfected into cultured cells or injected *in vivo*. Modified nucleotides (2′O methyl-ATP, N6 methyl-ATP, and pseudo-UTP) are introduced to increase the activity and stability of therapeutic SINEUPs. Structural studies using circular dichroism spectroscopy identified common core structures that govern the activity of miniSINEUPs with different modifications in solution that could further simplify their design and manufacturing (Valentini et al., 2022).

One such miniSINEUP, targeting human FXN mRNA, increased frataxin protein to physiological levels and promoted the recovery of disease-associated mitochondrial aconitase defects in Friedreich’s ataxia fibroblasts (Bon et al., 2019).

Another miniSINEUP targeting Gdnf mRNA delivered by an AAV9 construct increased endogenous Gdnf protein levels by about 2-fold *in vivo*, in the striatum of wild-type mice. Elevated levels of Gdnf protein persisted for at least 6 months. Furthermore, SINEUP-GDNF was able to ameliorate motor deficits and neurodegeneration of dopaminergic neurons in a neurochemical mouse model of Parkinson’s disease (Espinoza et al., 2020).

Other proposed applications of miniSINEUPs include upregulation of antibody production *in vitro* in CHO cells (Hoseinpoor et al., 2020), forecasting a promising future for this approach.

#### 3.2.2 MIR-NATs

NATs with embedded retrotransposon-derived repeats, such as mammalian-wide interspersed repeats (MIR), termed MIR-NATs, were also shown to form effector domains that regulate mRNA-ribosome pairing (Figure 3iii). MIR-NAT subclass of NATs may potentially overlap with SINEUPs.

In a disease-relevant example, MAPT-AS1 (microtubule-associated protein tau antisense 1) is a MIR-NAT overlapping head-to-head with MAPT 5′UTR. The overlap includes domain 2 of the MAPT-IRES that binds to 40S ribosomes. By competing for ribosomal pairing with MAPT mRNA, MAPT-AS1 repressed IRES-dependent translation of the MAPT gene product, protein tau. Overexpression of MAPT-AS1 or minimal essential sequences from MAPT-AS1 (including MIR) shifted MAPT mRNA from heavy to lighter polysomes and reduced tau levels in human induced pluripotent stem cell-derived neurons. NBT mimics of MAPT-AS minimal essential sequence may be therapeutically beneficial in tauopathies, Alzheimer’s and Parkinson’s disease (Grabowska-Pyrzewicz et al., 2021; Simone et al., 2021).

PLCG1 (phospholipase-C gamma 1), a possible alternative to amyloid-beta targeting in Alzheimer’s disease (AD), can be modulated via PLCG1-AS, a MIR-NAT with an inverted MIRb repeat. PLCG1-AS overlaps the 5′UTR of the PLCG1 gene and competes with PLCG1 5′UTR for binding to ribosomes, thus reducing PLCG1 protein expression (Simone et al., 2021).

#### 3.2.3 Regulation of miRNA activity

Multiple NATs have been shown to regulate translation and transcription of gene subsets or single genes through sponging endogenous miRNAs (Figure 3i). In some cases, knockdown of these NATs presents a desirable therapeutic target.

For example, KCNQ1OT1 (KCNQ1 opposite strand/antisense transcript 1) was induced in an *in vitro* model of diabetic nephropathy and promoted inflammation and apoptosis of podocytes by sponging miR-23b-3p and increasing expression of Sema3A, its direct target (Fei et al., 2022). Decreasing the levels of KCNQ1OT1 using NBTs may be useful in the treatment of diabetes and other inflammatory diseases.

Expression of protein components of biological clock coordinates multiple cellular processes and aligns them with circadian rhythms of the whole organism. Loss of such synchronization is thought to cause circadian rhythm disturbances in numerous diseases. PER2AS and CRY1AS are NATs expressed from the loci of the core circadian clock genes (PER2 and CRY1 respectively). Their expression is oscillating in phase with their partner coding genes in a 24-h period and upregulates corresponding mRNAs by blocking their binding to cognate miRNA (Najari Hanjani and Golalipour, 2021).

Interestingly, endogenous miRNA-sponging NATs can be delivered to target cells by exosomes. For instance, exosomes derived from endometrial stromal cells were shown to deliver AFAP1-AS1 (actin filament associated protein 1-antisense RNA 1) to other cells. AFAP1-AS1 blocks BCL9 (B-cell CLL/lymphoma 9) degradation by miR-15a-5p, facilitating cell migration and invasion. This effect was reversed by inhibition of exosomal AFAP1-AS1 in nude mice which could be beneficial in the treatment of cancers (Wang X. Y. et al., 2018; Wang et al., 2022).

NATs can also regulate miRNA activity by other mechanisms. For example, LYPLAL1-AS1 binds to promoter of microRNA let-7b gene (MIRLET7B) and represses its transcription. miR-let-7b is upregulated and LYPLAL1-AS1 is downregulated during senescence of human adipose-derived mesenchymal stem cells. Overexpression of LYPLAL1-AS1 attenuated senescence (Yang et al., 2022).

Using a different mechanism, interferon-α1 antisense RNA (IFN-α1AS) stabilizes its sense partner mRNA by sense-antisense duplex formation and inhibition of miR-1270-induced mRNA decay. Pulmonary-administered ASOs representing functional domains of guinea pig IFN-α1AS stabilized IFNA1 mRNA and inhibited influenza virus proliferation (Sakamoto et al., 2019).

As many NATs contain miRNA binding sites, synthetic anti-miRNA molecules (antimirs or RNA mimics) can be used to regulate their activity. In a disease-relevant example, a cluster of microRNA binding sites is present in the Ube3a1 NAT, including sites for miR-134. ICV injection of an antimir to miR-134 (Ant-134) reduced audiogenic seizure severity in Angelman syndrome (AS) mice carrying a maternal deletion of Ube3a (Ube3a m-/p+). Ant-134 also improved distance traveled and center crossings of AS mice in the open-field test, an approach being developed by Roche (Campbell et al., 2022).

#### 3.2.4 mRNA stability

One of the possible functions of NATs is to bind their mRNA partner, thereby modulating its half-life in a miRNA independent manner (Figure 3ii). This mechanism is utilized by Nqo1-AS1 (NAD(P)H dehydrogenase, quinone 1antisense 1, or Fantom3_F830212L20) that was mainly located in the cytoplasm of mouse alveolar epithelium cells and was increased after cigarette smoke exposure. Nqo1-AS1 overexpression attenuated oxidative stress by increasing mRNA and protein levels of Nqo1 and Serpina1 (serpin peptidase inhibitor, clade a, member 1) through antisense pairing with the Nqo1 3′UTR. ASO mimics of the duplex-forming domain of the Nqo1-AS1 may represent a therapeutic approach in chronic obstructive pulmonary disease (Zhang et al., 2021).

In another disease-relevant example, FAM83A-AS1 promoted lung adenocarcinoma progression by elevating FAM83A expression and activating ERK (extracellular signal-regulated kinase 1) signaling pathways. FAM83A-AS1 formed an RNA duplex with FAM83A mRNA thus increasing its stability and expression. Knockdown of FAM83A-AS1 can be achieved using NBTs (Wang W. et al., 2021a).

Transcript stability regulation can also be mediated by sponging protein factors that regulate degradation of mRNA. ADORA2A-AS1 (adenosine A2A receptor) bound HuR and thus prevented its binding to FSCN1 (fascin actin-bundling protein 1) transcripts, which decreased FSCN1 transcript stability and repressed AKT pathway activation. Low levels of ADORA2A-AS1 were correlated with poor prognosis in hepatocellular carcinoma (Pu et al., 2021).

Multiple NATs, such as VPS9D1-AS1, are involved in multiple regulatory pathways targeting disease-relevant protein(s). VPS9D1-AS1, expressed from the opposite strand in the VPS9D1 (VPS9 domain-containing protein 1) locus, bound HuR protein to regulate stability and expression of the CDK4 mRNA. VPS9D1-AS1 is overexpressed in hepatocellular carcinoma and multiple other cancers. VPS9D1-AS1 silencing suppressed hepatocellular carcinoma tumor growth *in vivo* (Zhou N. et al., 2021). VPS9D1-AS1 has also been shown to bind miR-520a-5p, hsa-miR-361-3p, miR-184 and several other miRNAs (Wang J. et al., 2018; Chen and Shen, 2022; Gao et al., 2022).

#### 3.2.5 Protein stability

Besides regulating stability of mRNAs, NATs are known to modulate protein stability (Figure 3vi). One of the mechanisms by which NATs enhance protein stability is blocking proteasomal degradation. For example, the antisense transcript KDM4A-AS1 binds to the androgen receptor (AR) protein and increases its stability by promoting deubiquitination of AR by scaffolding the USP14 (ubiquitin-specific protease 14)-AR complex, thus blocking AR degradation by the MDM2 (MDM2 protooncogene)-mediated ubiquitin-proteasome pathway. KDM4A-AS1 expression was elevated in castration-resistant prostate cancer. Importantly, ASOs targeting KDM4A-AS1 significantly reduced the growth of tumors with enzalutamide resistance (Zhang et al., 2022).

In another disease-relevant example, ARHGAP5-AS1 (NR_027263) interacted with SMAD7 (SMAD family member 7) through its PY motif and inhibited SMAD7 interaction with E3 ubiquitin ligases SMURF1 (SMAD-specific E3 ubiquitin protein ligase 1) and SMURF2. This led to SMAD7 protein stabilization and inhibition of breast cancer cell migration. NBT mimics of the SMAD7 interaction domain of ARHGAP5-AS1 could have therapeutic potential in breast cancer (Wang C. L. et al., 2021).

#### 3.2.6 Subcellular localization of proteins

NATs have also been reported to modulate subcellular distribution of proteins (Figure 3vii), although this aspect of their activity is not well studied at the moment. One example of this mechanism is panRNA-DMP1, a promoter-associated NAT from the dentin matrix protein-1 (DMP1) gene locus. PanRNA-DMP1 was induced by EGF (epidermal growth factor) stimulation. Depletion of panRNA-DMP1 increased EGFR (epidermal growth factor receptor) nuclear localization in response to EGF treatment and stabilized EGFR interactions with STAT3 (signal transducer and activator of transcription 3) resulting in enhanced cancer cell migration (Suzuki et al., 2021)^1^ ^,^ ^2^.

#### 3.2.7 NAT-encoded peptides

Interestingly, short ORFs present in some NATs can be translated into peptides that possess biological functions (Figure 3v). For example, HNF4A-AS1 (hepatocyte nuclear factor 4 alpha antisense RNA 1) encodes a 51-amino acid peptide (sPEP1). Translation of sPEP1 was facilitated by the interaction of HNF4A-AS1 and miRNA-409-5p via recruitment of EIF3G (eukaryotic translation initiation factor 3 subunit G). sPEP1 directly interacted with eEF1A1 (eukaryotic translation elongation factor 1 alpha 1) to stabilize its binding to tumor suppressor SMAD4 (SMAD family member 4), resulting in repression of SMAD4 transactivation. Expression of sPEP1 repressed serum deprivation-induced senescence and promoted growth and metastasis of neuroblastoma stem cells. Knockdown of HNF4A-AS1 or blocking sPEP1 translation may represent a therapeutic approach in relevant cancers (Song et al., 2022).

## 4 Multifunctional NATs and combinatorial NBTs

Accumulating data on the biological activity of NATs has established that each NAT can regulate protein expression through multiple mechanisms. For example, inhibition of APOA1-AS (apolipoprotein A-I antisense) expression resulted in upregulation of its sense partner gene APOA1 and of the two neighboring genes in the APO cluster by recruitment of lysine (K)-specific demethylase 1 (LSD1) and modulation of distinct histone methylation patterns (Halley et al., 2014). At the same time, APOA1-AS has been shown to recruit the TAF15 (TATA-box binding protein associated factor 15) protein to stabilize SMAD3 (SMAD family member 3) mRNA and activate the TGF-β/SMAD3 signaling pathway. Knockdown of APOA1-AS inhibited proliferation and migration and increased apoptosis in a cellular model of atherosclerosis (Wang J. et al., 2021a).

In another example, TRAF3IP2-AS1 (TRAF3-interacting protein 2 antisense 1) directly bound to PARP1 (poly (ADP-ribose) polymerase 1) mRNA and facilitated recruitment of the N6-methyladenosine methyltransferase complex, leading to accelerated decay of PARP1 mRNA. At the same time, TRAF3IP2-AS1, acting as a tumor suppressor, sequestered miR-200a-3p/153-3p/141-3p and prevented degradation of the tumor suppressor PTEN mediated by these miRNA (Yang L. et al., 2021).

ILF3-AS1 associates with ILF3 (interleukin enhancer-binding factor 3) mRNA and inhibits its degradation via recruiting m6A RNA methyltransferase METTL3 and enhancing ILF3 mRNA interactions with the m6A reader IGF2BP1. Knockdown of ILF3-AS1 resulted in the suppression of proliferation, migration and invasion in hepatocellular carcinoma cells (Bo et al., 2021). Furthermore, ILF3-AS1 accelerated the proliferation and metastasis of colorectal cancer cells by recruiting the histone methyltransferase EZH2 to induce trimethylation of H3K27 and downregulate the tumor suppressor CDKN2A (cyclin-dependent kinase inhibitor 2A) (Hong et al., 2021).

MUNC lncRNA (also known as DRReRNA) acts as an eRNA, maintaining open chromatin structure and inducing expression of the Myod1 (myoblast determination protein 1) gene in cis, and stimulating the expression of other promyogenic genes including Myog (myogenin) and Runx1 (runt-related transcription factor 1) in trans by recruiting the cohesin complex to their promoters (Przanowska et al., 2022). MUNC can also block binding of transcriptional enhancers and inhibit transcription from other gene promoters, such as Il15 (interleukin-15), Prmt6 (protein arginine methyltransferase 6), and Grip1 (glutamate receptor-interacting protein 1) possibly orchestrating multiple aspects of the myogenic process (Przanowska et al., 2022).

Many of the multiplex NAT functions could be independently and specifically modulated by distinct NBTs. However, in cases where different aspects of NAT functions regulate the same disease-relevant pathway it could be beneficial to design NBTs that simultaneously affect multiple functionalities. In one example of this combinatorial approach, ASOs inhibiting the activity of SMN-AS1, known to repress SMN1 transcription by recruiting PRC2 (polycomb repressive complex 2), delivered together with SMN2 splice-switching ASOs synergistically increased SMN protein expression and improved survival of a mouse model of severe SMA. This approach is investigated by Ionis (d'Ydewalle et al., 2017). In another example, combined treatment with ASOs targeted against key methylation sites in the SMN2 promoter region and ASOs enhancing exon 7 retention in SMN2 mRNA resulted in a synergistic effect on functional SMN protein levels (Wang J. et al., 2021b).

However, as many ASOs show a cumulative bell-shaped dose response curve, delivering multiple ASOs at the same time may not be practical. For example, treatment of SMA fibroblasts with three ASOs targeting ISS-N1 site in intron 7, and 3′ splice site and 5′ region of exon 8 respectively did not significantly increase the production of functional SMN protein. At the same time, as combined ASO dosage increases, the probability of toxic side effects due to kidney and liver overload becomes higher. Furthermore, treatment with a mixture of two different ASOs resulted in inclusion of a novel cryptic exon in the SMN protein (Wijaya et al., 2022). Addition of a degradable chemical linker between two or more ASOs or siRNAs has been proposed (Alterman et al., 2019). Such linkers can be engineered to release the oligonucleotide payload in specific tissues. For example, Val-Ala-02 and Val-Ala-Chalcone linkers are preferentially digested by cathepsin B, which is highly expressed in the brain, enabling *in situ* cleavage of linked oligonucleotides into separate active components (Jin et al., 2022). Vectorized delivery of multiple NBTs is a possible way to mitigate the high oligonucleotide dosage problem. In this case the active NBTs are expressed by a viral or plasmid vector within the target cell and do not overload the uptake and clearance mechanisms, thus reducing the possibility of adverse events.

## 5 Regulation of NAT expression

One of the areas essential for NAT-targeted therapeutic development that remains poorly studied is the regulation and expression of NATs themselves. In the emerging picture, most of the mechanisms known to regulate expression of protein-coding genes, pRNAs and eRNAs are involved in modulating NAT expression. Interestingly, this also includes NAT-mediated regulation of expression of other NATs. Therefore, most of the NBT approaches described above could be applicable to regulation of disease-relevant NATs that could be employed instead of introducing NAT mimics.

It has been shown that NAT expression can be subject to imprinting induced by environmental factors. For example, maternal obesity in mice leads to maternal imprinting and reduced expression of a deiodinase 3 (Dio3) NAT (Dio3os) in fetal brown fat. The resulting enhanced expression of Dio3, which catabolizes triiodothyronine (T3), leads to intracellular T3 deficiency, suppression of brown adipose tissue development and obesity in the offspring. NBTs that could recruit DNA demethylases to Dio3os promoter may reverse this condition (Chen et al., 2021).

Long range promoter-enhancer interaction can play a role in regulation of NAT expression. It has been shown that the A allele of the SNP rs2647046 located in an intergenic enhancer in the HLA (human leukocyte antigen) locus leads to increased transcription of HLA-DQB1-AS1 via facilitating CTCF-mediated long-range loop formation, enhancer-promoter interaction and transcription factor binding. The rs2647046 A allele is associated with increased risk of hepatocellular carcinoma. NBTs editing or blocking the effects of the A allele could permit a minimally disruptive intervention in this cancer (Wang H. et al., 2021).

Epigenetic DNA methylation of promoters is also involved in the regulation of NAT expression. ZNF582-AS1 is downregulated in clear cell renal cell carcinoma involving DNA methylation at the CpG islands within its promoter (Yang W. et al., 2021).

NAT expression can be affected by the same transcription factors that regulate expression of mRNA. For example, active vitamin D receptor (VDR) binds to consensus VDR-binding motifs in NAT genes. One of the VDR-regulated NATs is AS-HSD17b2, that is transcribed from the antisense strand of the 17-beta-hydroxysteroid dehydrogenase type 2 (HSD17b2) locus. Upregulation of AS-HSD17b2 inhibits HSD17b2 expression (Kanemoto et al., 2022).

NAT transcription can be activated by Ets/TCF transcription factor family members, such as GABP (GA-binding protein transcription factor, beta subunit) and Ets1/2 (ETS protooncogene 1, transcription factor). Such activation is observed in an interesting case of oncogenic mutations −124C>T and −146C>T in the bidirectional promoter that drives expression of TERT (telomerase catalytic subunit, telomerase reverse transcriptase) and its partner NAT TAPAS. These mutations create novel Ets/TCF binding sites (GGAA, or TTCC on the opposite strand) in the promoter, which leads to Ets/TCF factor binding and upregulation of TAPAS expression (Hafezi et al., 2021).

Protein products of their partner coding genes are also known to regulate NAT expression. The HIF-1α (hypoxia-inducible factor 1, alpha subunit) NAT (HIFAL) recruits prolyl hydroxylase 3 (PHD3) to pyruvate kinase 2 (PKM2) protein to induce its prolyl hydroxylation. HIFAL also facilitates the transport of the PKM2/PHD3 complex into the nucleus by binding hnRNPF (heterogeneous nuclear ribonucleoprotein F), which leads to enhanced expression of HIF-1α. Moreover, HIF-1α protein induces HIFAL transcription thus forming a positive feed-back loop. High HIFAL expression is associated with an aggressive breast cancer phenotype (Zheng et al., 2021).

In a “cross-loci” feed-back example, HIF-1α bound to a hypoxia response element in the ZEB1-AS1 promoter and upregulated its expression. ZEB1-AS1, in turn, upregulated expression of ZEB1, a transcription factor that induces epithelial-mesenchymal transition and plays a crucial role in the progression of cancers. Furthermore, ZEB1-AS1 scaffolded the interaction of HIF-1α, ZEB1, and HDAC1, leading to deacetylation and stabilization of HIF-1α protein, thus forming a positive feedback loop. Knockdown of ZEB1-AS1 inhibited progression and metastasis of pancreatic cancer in nude mice (Jin et al., 2021).

NAT stability can also be actively regulated through post-transcriptional modification and modulation of miRNA and protein binding. For example, BDNF-AS expression is increased in response to decreased levels of N6-methyladenosine on BDNF-AS. Upregulation of BDNF-AS is associated with decreased BDNF expression (Bohnsack et al., 2019). While the promoter of OSER1-AS1 (oxidative stress responsive serine rich 1 antisense 1) can be repressed by MYC, the 3′-end of OSER1-AS1 is competitively targeted by microRNA hsa-miR-17-5p and RNA-stabilizing protein HuR (Xie W. et al., 2021).

## 6 Clinical experience with RNA-targeting therapies

The critical question for the NBT field in general, and NAT-targeted therapies in particular, is whether the initial successes achieved in recent years would lead to lasting development. To a large extent this will be determined by safety, efficacy and commercial viability of the NBTs. This was demonstrated by mipomersen, that was discontinued due to liver toxicity and also pegaptanib, which was abandoned by the manufacturer for poor commercial performance (Table 1).

As nusinersen is the last generation NBT with the longest history of clinical use, and as the chemistry, PK and PD of ASO drugs have proven to be similar, nusinersen results could forecast the clinical experience with other ASOs and future prospects of the NBT field in general.

Nusinersen (SPINRAZA®) was approved by the FDA in 2016 for the treatment of spinal muscular atrophy (SMA). As described above, nusinersen increases expression of functional SMN protein for the treatment of SMA. Nusinersen is administered intrathecally, starting with three loading doses at 2-weeks intervals, followed by a fourth loading dose 30 days later and maintenance dosing at 4-months intervals.

Reports on long-term efficacy and safety of nusinersen have been encouraging. Meta-analysis of more than 20 clinical studies evaluating motor function in type 2 and 3 SMA patients treated with nusinersen revealed consistently positive outcomes (Coratti et al., 2021). Review of 271 SMA patients showed motor function improvements in 26.2% and overall improvement in 99.6–100.0% of patients treated with nusinersen in Japan between 2017 and 2019 (Wataya et al., 2021).

Good safety record of nusinersen over more than 5 years in the clinic is particularly notable because of its IT route of administration that potentially is an independent source of adverse events, especially in SMA where scoliosis and osseous fusion are frequent. In fact, most commonly reported adverse events during treatment with nusinersen were consistent with symptoms of SMA and lumbar puncture (Stolte et al., 2021; Wataya et al., 2021). Notably, retrospective claims database analysis of more than 300 SMA patients for years 2016–2019 indicated that fewer than 50% of patients who completed the loading phase of nusinersen treatment remained in treatment 24 months after start, likely due to logistical and medical challenges of the IT administration route, which highlights the importance of a non-invasive route of administration for wide acceptance of NBTs (Gauthier-Loiselle et al., 2021).

Reports on the efficacy and safety of other FDA-approved NBTs are also encouraging. Eteplirsen (EXONDYS 51®), golodirsen (VYONDYS 53™), viltolarsen (VILTEPSO®) and casimersen (Amondys 45) are clinically approved splice-switching antisense oligonucleotides to treat Duchenne muscular dystrophy (DMD) associated with exon 51, 53 or 45 mutations (Table 1). Due to the molecular structure of dystrophin, skipping these exons does not significantly affect protein function. Although initial approval of eteplirsen was controversial due to poor evidence of efficacy at the time, evaluation of treatment effects of eteplirsen over a 6 years-follow-up period have demonstrated that eteplirsen-treated patients (*n* = 22) experienced longer median time to loss of ambulation (+2.09 years, *p* < 0.01) and attenuated rates of pulmonary decline (*p* < 0.0001) compared to standard-of-care external controls (Mitelman et al., 2022). In an encouraging further development, clinical trials showed improved performance of SRP-5051, a next-generation peptide-conjugated phosphorodiamidate morpholino oligomer (PPMO) candidate for exon 51 skipping in DMD. Positive clinical results have also been reported for golodirsen and viltolarsen (Servais et al., 2022).

Clinical experience with NBTs also elucidates possible adverse events that could be common to NBT as a class. Most frequent NBT-associated adverse events are related to NBT delivery methods. Injection site reactions of different severity are observed for all current administration techniques, with more severe ones being associated with intraocular, intracerebroventricular and intrathecal routes. Furthermore, flu-like symptoms, such as fatigue, influenza-like illness, chills, pyrexia, arthralgia, myalgia, or malaise were reported. Other class AE for oligonucleotide NBTs may include liver and kidney toxicity, as ASOs are preferentially accumulated in these organs. Some of the oligonucleotide drug-treated patients developed thrombocytopenia. Golodirsen-treated patients exhibited hypersensitivity reactions, including rash and skin exfoliation (Alhamadani et al., 2022).

Encouragingly, the accumulation of clinical and animal testing data combined with data mining allow for the development of in-silico safety pre-screening and identification of toxicity screens that are highly predictive of patient outcomes. Importantly, with each new generation of NBTs there has been improvement in their safety and tolerability, especially in kidney and liver toxicity and flu-like symptoms (Partridge et al., 2021).

## 7 Conclusion and future outlook

Recent discoveries in genomics and proteomics have shed light on the central role of ncRNA-based mechanisms in regulating protein expression. This has opened a vast new set of therapeutic targets that historically were inaccessible to traditional protein-targeted small molecule inhibitors. Furthermore, technological progress in NBT chemistry and biology has provided a convenient tool for modulating RNA-based mechanisms. Importantly, more streamlined design and development procedures of NBTs make them perfectly suited for the treatment of rare diseases and subsets of ‘common’ diseases associated with particular genes and for the goals of personalized medicine.

As more information on NBTs in the clinic is available, it is becoming clear that, in spite of unprecedented clinical successes in treating some severe disorders, the current NBT technologies do not lead to a complete cure. Therefore, further studies of the genetic determinants and pathophysiology of diseases are essential for better identification of NBT targets and for improving treatment regimens. Combinatorial approaches, delivering mixes of NBTs or NBTs and small molecules that target different but complementary biological pathways may further improve treatment outcomes. The development of early diagnostic techniques would allow early treatment initiation crucial in genetic disorders and fast developing cancers.

Furthermore, it is obvious from the clinical experience that less invasive and more convenient administration routes are necessary for the NAT-targeting NBTs to become widely adopted. Recent clinical trials with NBT administration through inhalation and work on oral, intranasal and MIND techniques is encouraging, but significant further effort is needed.

Overall, in spite of setbacks in the NAT-targeting field described above, the experience of companies that are actively working to improve NBT technology has been fruitful and demonstrates progress toward better treatment outcomes.

## Footnotes

1 Sarepta Therapeutics Reports Positive Clinical Results from Phase 2 MOMENTUM Study of SRP-5051 in Patients with Duchenne Muscular Dystrophy Amenable to Skipping Exon 51. https://investorrelations.sarepta.com/ news-releases/news-release-details/sarepta- therapeutics-reports-positive-clinical-results-phase-2 (2021).

2 VILTEPSO® (viltolarsen) injection: Long-Term Efficacy and Safety Data Presented at the PPMD 2021 Virtual Annual Conference. Nippon Shinyaki https://www.nippon-shinyaku.co.jp/file/download.php?file_id=5195 (2021).

## References

[B147] Agostini F. Zagalak J. Attig J. Ule J. Luscombe N. M. (2021). Intergenic RNA mainly derives from nascent transcripts of known genes. Genome. Biol. 22 (1), 136. 10.1186/s13059-021-02350-x 33952325 PMC8097831

[B1] Alhamadani F. Zhang K. Parikh R. Wu H. Rasmussen T. P. Bahal R. (2022). Adverse drug reactions and toxicity of the Food and drug administration-approved antisense oligonucleotide drugs. Drug Metab. Dispos. 50, 879–887. 10.1124/dmd.121.000418 35221289 PMC11022857

[B2] Alterman J. F. Godinho B. M. D. C. Hassler M. R. Ferguson C. M. Echeverria D. Sapp E. (2019). A divalent siRNA chemical scaffold for potent and sustained modulation of gene expression throughout the central nervous system. Nat. Biotechnol. 37 (8), 884–894. 10.1038/s41587-019-0205-0 31375812 PMC6879195

[B3] Andergassen D. Rinn J. L. (2022). From genotype to phenotype: Genetics of mammalian long non-coding RNAs *in vivo* . Nat. Rev. Genet. 23 (4), 229–243. 10.1038/s41576-021-00427-8 34837040

[B4] Andergassen D. Smith Z. D. Kretzmer H. Rinn J. L. Meissner A. (2021). Diverse epigenetic mechanisms maintain parental imprints within the embryonic and extraembryonic lineages. Dev. Cell 56 (21), 2995–3005.e4. e4. 10.1016/j.devcel.2021.10.010 34752748 PMC9463566

[B5] Arnoldi M. Zarantonello G. Espinoza S. Gustincich S. Di Leva F. Biagioli M. (2022). Design and delivery of SINEUP: A new modular tool to increase protein translation. Methods Mol. Biol. 2434, 63–87. 10.1007/978-1-0716-2010-6_4 35213010 PMC9703201

[B6] Barbier A. J. Jiang A. Y. Zhang P. Wooster R. Anderson D. G. (2022). The clinical progress of mRNA vaccines and immunotherapies. Nat. Biotechnol. 40 (6), 840–854. 10.1038/s41587-022-01294-2 35534554

[B7] Bellido Molias F. Sim A. Leong K. W. An O. Song Y. Ng V. H. E. (2021). Antisense RNAs influence promoter usage of their counterpart sense genes in cancer. Cancer Res. 81 (23), 5849–5861. 10.1158/0008-5472.CAN-21-1859 34649947 PMC9397637

[B8] Blank-Giwojna A. Postepska-Igielska A. Grummt I. (2019). lncRNA KHPS1 activates a poised enhancer by triplex-dependent recruitment of epigenomic regulators. Cell Rep. 26 (11), 2904–2915. e4. 10.1016/j.celrep.2019.02.059 30865882

[B9] Bo C. Li N. He L. Zhang S. An Y. (2021). Long non-coding RNA ILF3-AS1 facilitates hepatocellular carcinoma progression by stabilizing ILF3 mRNA in an m6A-dependent manner. Hum. Cell 34 (6), 1843–1854. 10.1007/s13577-021-00608-x 34491544

[B10] Bohnsack J. P. Teppen T. Kyzar E. J. Dzitoyeva S. Pandey S. C. (2019). The lncRNA BDNF-AS is an epigenetic regulator in the human amygdala in early onset alcohol use disorders. Transl. Psychiatry 9 (1), 34. 10.1038/s41398-019-0367-z 30728347 PMC6365546

[B11] Bon C. Luffarelli R. Russo R. Fortuni S. Pierattini B. Santulli C. (2019). SINEUP non-coding RNAs rescue defective frataxin expression and activity in a cellular model of Friedreich's Ataxia. Nucleic Acids Res. 47 (20), 10728–10743. 10.1093/nar/gkz798 31584077 PMC6847766

[B12] Brown K. M. Nair J. K. Janas M. M. Anglero-Rodriguez Y. I. Dang L. T. H. Peng H. (2022). Expanding RNAi therapeutics to extrahepatic tissues with lipophilic conjugates. Nat. Biotechnol. 10.1038/s41587-022-01334-x 35654979

[B13] Bull S. C. Doig A. J. (2015). Properties of protein drug target classes. PLoS One 10 (3), e0117955. 10.1371/journal.pone.0117955 25822509 PMC4379170

[B14] Campbell A. Morris G. Sanfeliu A. Augusto J. Langa E. Kesavan J. C. (2022). AntimiR targeting of microRNA-134 reduces seizures in a mouse model of Angelman syndrome. Mol. Ther. Nucleic Acids 28, 514–529. 10.1016/j.omtn.2022.04.009 35592499 PMC9092865

[B15] Che Y. Lin Y. Shu Y. He J. Gao W. (2020). Interaction between N6-methyladenosine (m6A) modification and noncoding RNAs in cancer. Mol. Cancer 19 (1), 94. 10.1186/s12943-020-01207-4 32443966 PMC7243333

[B16] Chen L. Shen M. (2022). LncRNA VPS9D1-AS1 sponging miR-520a-5p contributes to the development of uterine corpus endometrial carcinoma by enhancing BIRC5 expression. Mol. Biotechnol. 10.1007/s12033-022-00510-3 35619019

[B17] Chen W. Hu Y. Ju D. (2020). Gene therapy for neurodegenerative disorders: Advances, insights and prospects. Acta Pharm. Sin. B 10 (8), 1347–1359. 10.1016/j.apsb.2020.01.015 32963936 PMC7488363

[B18] Chen Y. T. Yang Q. Y. Hu Y. Liu X. D. de Avila J. M. Zhu M. J. (2021). Imprinted lncRNA Dio3os preprograms intergenerational Brown fat development and obesity resistance. Nat. Commun. 12 (1), 6845. 10.1038/s41467-021-27171-1 34824246 PMC8617289

[B19] Cini G. Quaia M. Canzonieri V. Fornasarig M. Maestro R. Morabito A. (2019). Toward a better definition of EPCAM deletions in Lynch Syndrome: Report of new variants in Italy and the associated molecular phenotype. Mol. Genet. Genomic Med. 7 (5), e587. 10.1002/mgg3.587 30916491 PMC6503020

[B20] Coratti G. Cutrona C. Pera M. C. Bovis F. Ponzano M. Chieppa F. (2021). Motor function in type 2 and 3 SMA patients treated with nusinersen: A critical review and meta-analysis. Orphanet J. Rare Dis. 16 (1), 430. 10.1186/s13023-021-02065-z 34645478 PMC8515709

[B21] Crooke S. T. Baker B. F. Crooke R. M. Liang X. H. (2021). Antisense technology: An overview and prospectus. Nat. Rev. Drug Discov. 20 (6), 427–453. 10.1038/s41573-021-00162-z 33762737

[B22] d'Ydewalle C. Ramos D. M. Pyles N. J. Ng S. Y. Gorz M. Pilato C. M. (2017). The antisense transcript SMN-AS1 regulates SMN expression and is a novel therapeutic target for spinal muscular atrophy. Neuron 93 (1), 66–79. 10.1016/j.neuron.2016.11.033 28017471 PMC5223741

[B23] Espinoza S. Scarpato M. Damiani D. Managò F. Mereu M. Contestabile A. (2020). SINEUP non-coding RNA targeting GDNF rescues motor deficits and neurodegeneration in a mouse model of Parkinson's disease. Mol. Ther. 28 (2), 642–652. 10.1016/j.ymthe.2019.08.005 31495777 PMC7000958

[B24] Fallatah B. Shuaib M. Adroub S. Paytuví-Gallart A. Della Valle F. Nadeef S. (2021). Ago1 controls myogenic differentiation by regulating eRNA-mediated CBP-guided epigenome reprogramming. Cell Rep. 37 (9), 110066. 10.1016/j.celrep.2021.110066 34852230

[B25] Fasolo F. Patrucco L. Volpe M. Bon C. Peano C. Mignone F. (2019). The RNA-binding protein ILF3 binds to transposable element sequences in SINEUP lncRNAs. FASEB J. 33 (12), 13572–13589. 10.1096/fj.201901618RR 31570000 PMC6894054

[B26] Fei B. Zhou H. He Z. Wang S. (2022). KCNQ1OT1 inhibition alleviates high glucose-induced podocyte injury by adsorbing miR-23b-3p and regulating Sema3A. Clin. Exp. Nephrol. 26, 385–397. 10.1007/s10157-021-02173-x 34997887

[B27] Fu W. Ren H. Shou J. Liao Q. Li L. Shi Y. (2022). Loss of NPPA-AS1 promotes heart regeneration by stabilizing SFPQ-NONO heteromer-induced DNA repair. Basic Res. Cardiol. 117 (1), 10. 10.1007/s00395-022-00921-y 35247074

[B28] Gao X. Zhang S. Wang X. (2022). VPS9D1-AS1 gene rs7206570 polymorphism associated with the clinical stage of colorectal cancer and binding with hsa-miR-361-3p. Hum. Cell 35 (2), 522–527. 10.1007/s13577-021-00658-1 35022999

[B29] Gauthier-Loiselle M. Cloutier M. Toro W. Patel A. Shi S. Davidson M. (2021). Nusinersen for spinal muscular atrophy in the United States: Findings from a retrospective claims database analysis. Adv. Ther. 38 (12), 5809–5828. 10.1007/s12325-021-01938-w 34713391 PMC8552979

[B30] Gennemark P. Walter K. Clemmensen N. Rekić D. Nilsson C. A. M. Knöchel J. (2021). An oral antisense oligonucleotide for PCSK9 inhibition. Sci. Transl. Med. 13 (593), eabe9117. 10.1126/scitranslmed.abe9117 33980578

[B31] Gorbovytska V. Kim S. K. Kuybu F. Götze M. Um D. Kang K. (2022). Enhancer RNAs stimulate Pol II pause release by harnessing multivalent interactions to NELF. Nat. Commun. 13 (1), 2429. 10.1038/s41467-022-29934-w 35508485 PMC9068813

[B32] Grabowska-Pyrzewicz W. Want A. Leszek J. Wojda U. (2021). Antisense oligonucleotides for alzheimer's disease therapy: From the mRNA to miRNA paradigm. EBioMedicine 74, 103691. 10.1016/j.ebiom.2021.103691 34773891 PMC8602003

[B33] Greulich F. Bielefeld K. A. Scheundel R. Mechtidou A. Strickland B. Uhlenhaut N. H. (2021). Enhancer RNA expression in response to glucocorticoid treatment in murine macrophages. Cells 11 (1), 28. 10.3390/cells11010028 35011590 PMC8744892

[B34] Guéant J. L. Siblini Y. Chéry C. Schmitt G. Guéant-Rodriguez R. M. Coelho D. (2022). Epimutation in inherited metabolic disorders: The influence of aberrant transcription in adjacent genes. Hum. Genet. 141, 1309–1325. 10.1007/s00439-021-02414-9 35190856

[B35] Hafezi F. Jaxel L. Lemaire M. Turner J. D. Perez-Bercoff D. (2021). TERT promoter mutations increase sense and antisense transcription from the TERT promoter. Biomedicines 9 (12), 1773. 10.3390/biomedicines9121773 34944589 PMC8698883

[B36] Halley P. Kadakkuzha B. M. Faghihi M. A. Magistri M. Zeier Z. Khorkova O. (2014). Regulation of the apolipoprotein gene cluster by a long noncoding RNA. Cell Rep. 6 (1), 222–230. 10.1016/j.celrep.2013.12.015 24388749 PMC3924898

[B37] Hashimoto A. Sarker D. Reebye V. Jarvis S. Sodergren M. H. Kossenkov A. (2021). Upregulation of C/EBPα inhibits suppressive activity of myeloid cells and potentiates antitumor response in mice and patients with cancer. Clin. Cancer Res. 27 (21), 5961–5978. 10.1158/1078-0432.CCR-21-0986 34407972 PMC8756351

[B38] He Y. Yue H. Cheng Y. Ding Z. Xu Z. Lv C. (2021). ALKBH5-mediated m6A demethylation of KCNK15-AS1 inhibits pancreatic cancer progression via regulating KCNK15 and PTEN/AKT signaling. Cell Death Dis. 12 (12), 1121. 10.1038/s41419-021-04401-4 34853296 PMC8636648

[B39] Hon C. C. Ramilowski J. A. Harshbarger J. Bertin N. Rackham O. J. Gough J. (2017). An atlas of human long non-coding RNAs with accurate 5' ends. Nature 543 (7644), 199–204. 10.1038/nature21374 28241135 PMC6857182

[B40] Hong S. Li S. Bi M. Yu H. Yan Z. Liu T. (2021). lncRNA ILF3-AS1 promotes proliferation and metastasis of colorectal cancer cells by recruiting histone methylase EZH2. Mol. Ther. Nucleic Acids 24, 1012–1023. 10.1016/j.omtn.2021.04.007 34141456 PMC8167202

[B41] Hopkins A. L. Groom C. R. (2002). The druggable genome. Nat. Rev. Drug Discov. 1, 727–730. 10.1038/nrd892 12209152

[B42] Hoseinpoor R. Kazemi B. Rajabibazl M. Rahimpour A. (2020). Improving the expression of anti-IL-2Rα monoclonal antibody in the CHO cells through optimization of the expression vector and translation efficiency. J. Biotechnol. 324, 112–120. 10.1016/j.jbiotec.2020.09.006 33007349

[B43] Hsiao J. Yuan T. Y. Tsai M. S. Lu C. Y. Lin Y. C. Lee M. L. (2016). Upregulation of haploinsufficient gene expression in the brain by targeting a long non-coding RNA improves seizure phenotype in a model of Dravet syndrome. EBioMedicine 9, 257–277. 10.1016/j.ebiom.2016.05.011 27333023 PMC4972487

[B44] Isoda T. Moore A. J. He Z. Chandra V. Aida M. Denholtz M. (2017). Non-coding transcription instructs chromatin folding and compartmentalization to dictate enhancer-promoter communication and T cell fate. Cell 171 (1), 103–119. e18. 10.1016/j.cell.2017.09.001 28938112 PMC5621651

[B45] Jin C. Ei-Sagheer A. H. Li S. Vallis K. A. Tan W. Brown T. (2022). Engineering enzyme-cleavable oligonucleotides by automated solid-phase incorporation of cathepsin B sensitive dipeptide linkers. Angew. Chem. Int. Ed. Engl. 61 (13), e202114016. 10.1002/anie.202114016 34953094 PMC9306542

[B46] Jin Y. Zhang Z. Yu Q. Zeng Z. Song H. Huang X. (2021). Positive reciprocal feedback of *lncRNA ZEB1-AS1* and *HIF-1*α contributes to hypoxia-promoted tumorigenesis and metastasis of pancreatic cancer. Front. Oncol. 11, 761979. Erratum in: Front Oncol. 2021;11:821077. 10.3389/fonc.2021.761979 34881179 PMC8645903

[B47] Joshi R. Salton S. R. J. (2022). Neurotrophin crosstalk in the etiology and treatment of neuropsychiatric and neurodegenerative disease. Front. Mol. Neurosci. 15, 932497. 10.3389/fnmol.2022.932497 35909451 PMC9335126

[B48] Kanemoto Y. Nishimura K. Hayakawa A. Sawada T. Amano R. Mori J. (2022). A long non-coding RNA as a direct vitamin D target transcribed from the anti-sense strand of the human HSD17B2 locus. Biosci. Rep. 42, BSR20220321. 10.1042/BSR20220321 35510872 PMC9142830

[B49] Khorkova O. Hsiao J. Wahlestedt C. (2021). Nucleic acid-based therapeutics in orphan neurological disorders: Recent developments. Front. Mol. Biosci. 8, 643681. 10.3389/fmolb.2021.643681 33996898 PMC8115123

[B50] Khorkova O. Wahlestedt C. (2017). Oligonucleotide therapies for disorders of the nervous system. Nat. Biotechnol. 35 (3), 249–263. 10.1038/nbt.3784 28244991 PMC6043900

[B51] Kim B. Luo Y. Zhan X. Zhang Z. Shi X. Yi J. (2021). Neuronal activity-induced BRG1 phosphorylation regulates enhancer activation. Cell Rep. 36 (2), 109357. 10.1016/j.celrep.2021.109357 34260936 PMC8315893

[B52] Lam M. T. Li W. Rosenfeld M. G. Glass C. K. (2014). Enhancer RNAs and regulated transcriptional programs. Trends biochem. Sci. 39 (4), 170–182. 10.1016/j.tibs.2014.02.007 24674738 PMC4266492

[B53] Lan T. Li H. Zhang D. Xu L. Liu H. Hao X. (2019). KIAA1429 contributes to liver cancer progression through N6-methyladenosine-dependent post-transcriptional modification of GATA3. Mol. Cancer 18 (1), 186. 10.1186/s12943-019-1106-z 31856849 PMC6921542

[B54] Lanciano S. Cristofari G. (2020). Measuring and interpreting transposable element expression. Nat. Rev. Genet. 21 (12), 721–736. 10.1038/s41576-020-0251-y 32576954

[B55] Lee J. H. Wang R. Xiong F. Krakowiak J. Liao Z. Nguyen P. T. (2021). Enhancer RNA m6A methylation facilitates transcriptional condensate formation and gene activation. Mol. Cell 81 (16), 3368–3385.e9. e9. 10.1016/j.molcel.2021.07.024 34375583 PMC8383322

[B56] Lewis M. W. Wisniewska K. King C. M. Li S. Coffey A. Kelly M. R. (2022). Enhancer RNA transcription is essential for a novel CSF1 enhancer in triple-negative breast cancer. Cancers (Basel) 14 (7), 1852. 10.3390/cancers14071852 35406623 PMC8997997

[B57] Li C. Gui G. Zhang L. Qin A. Zhou C. Zha X. (2022). Overview of methionine adenosyltransferase 2A (MAT2A) as an anticancer target: Structure, function, and inhibitors. J. Med. Chem. 65 (14), 9531–9547. 10.1021/acs.jmedchem.2c00395 35796517

[B58] Li L. C. Okino S. T. Zhao H. Pookot D. Place R. F. Urakami S. (2006). Small dsRNAs induce transcriptional activation in human cells. Proc. Natl. Acad. Sci. U. S. A. 103 (46), 17337–17342. 10.1073/pnas.0607015103 17085592 PMC1859931

[B59] Li W. Notani D. Rosenfeld M. G. (2016). Enhancers as non-coding RNA transcription units: Recent insights and future perspectives. Nat. Rev. Genet. 17 (4), 207–223. 10.1038/nrg.2016.4 26948815

[B60] Li Y. Syed J. Sugiyama H. (2016). RNA-DNA triplex formation by long noncoding RNAs. Cell Chem. Biol. 23 (11), 1325–1333. 10.1016/j.chembiol.2016.09.011 27773629

[B61] Liu X. Guo Z. Han J. Peng B. Zhang B. Li H. (2022). The PAF1 complex promotes 3' processing of pervasive transcripts. Cell Rep. 38 (11), 110519. 10.1016/j.celrep.2022.110519 35294889

[B62] Liu Y. Williams S. G. Jones H. R. Keavney B. D. Choy M. K. (2021). A novel RNA-mediated mechanism causing down-regulation of insulating promoter interactions in human embryonic stem cells. Sci. Rep. 11 (1), 23233. 10.1038/s41598-021-02373-1 34853328 PMC8636647

[B63] Ma M. Xiong W. Hu F. Deng M. F. Huang X. Chen J. G. (2020). A novel pathway regulates social hierarchy via lncRNA AtLAS and postsynaptic synapsin IIb. Cell Res. 30 (2), 105–118. 10.1038/s41422-020-0273-1 31959917 PMC7015055

[B64] Mahboobeh Z. Pegah M. Fatemeh S. Elham K. Hanieh A. Milad R. (2020). lncRNA ZEB2-AS1: A promising biomarker in human cancers. IUBMB Life 72 (9), 1891–1899. 10.1002/iub.2338 32687675

[B65] Mancini M. A. Li W. Xiong F. Krakowiak J. Liao Z. Nguyen P. T. (2021). Enhancer RNA m6A methylation facilitates transcriptional condensate formation and gene activation. Mol. Cell 81 (16), 3368–3385.e9. e9. 10.1016/j.molcel.2021.07.024 34375583 PMC8383322

[B66] Matsui M. Sakurai F. Elbashir S. Foster D. J. Manoharan M. Corey D. R. (2010). Activation of LDL receptor expression by small RNAs complementary to a noncoding transcript that overlaps the LDLR promoter. Chem. Biol. 17 (12), 1344–1355. 10.1016/j.chembiol.2010.10.009 21168770 PMC3071588

[B67] Melo C. A. Drost J. Wijchers P. J. van de Werken H. de Wit E. Oude Vrielink J. A. (2013). eRNAs are required for p53-dependent enhancer activity and gene transcription. Mol. Cell 49 (3), 524–535. 10.1016/j.molcel.2012.11.021 23273978

[B68] Milazzo C. Mientjes E. J. Wallaard I. Rasmussen S. V. Erichsen K. D. Kakunuri T. (2021). Antisense oligonucleotide treatment rescues UBE3A expression and multiple phenotypes of an Angelman syndrome mouse model. JCI Insight 6 (15), e145991. 10.1172/jci.insight.145991 PMC8410092 34369389

[B69] Mitelman O. Abdel-Hamid H. Z. Byrne B. J. Connolly A. M. Heydemann P. Proud C. (2022). A combined prospective and retrospective comparison of long-term functional outcomes suggests delayed loss of ambulation and pulmonary decline with long-term eteplirsen treatment. J. Neuromuscul. Dis. 9 (1), 39–52. 10.3233/JND-210665 34420980 PMC8842766

[B70] Modarresi F. Faghihi M. A. Lopez-Toledano M. A. Fatemi R. P. Magistri M. Brothers S. P. (2012). Inhibition of natural antisense transcripts *in vivo* results in gene-specific transcriptional upregulation. Nat. Biotechnol. 30 (5), 453–459. 10.1038/nbt.2158 22446693 PMC4144683

[B71] Morrissy A. S. Griffith M. Marra M. A. (2011). Extensive relationship between antisense transcription and alternative splicing in the human genome. Genome Res. 21 (8), 1203–1212. 10.1101/gr.113431.110 21719572 PMC3149488

[B72] Nair S. J. Suter T. Wang S. Yang L. Yang F. Rosenfeld M. G. (2022). Transcriptional enhancers at 40: Evolution of a viral DNA element to nuclear architectural structures. Trends Genet. S0168-9525(22)00141-X. 10.1016/j.tig.2022.05.015 PMC9474616 35811173

[B73] Najari Hanjani P. Golalipour M. (2021). Circadian oscillation of natural antisense transcripts related to human core clock genes. Rep. Biochem. Mol. Biol. 10 (3), 471–476. 10.52547/rbmb.10.3.471 34981025 PMC8718779

[B74] Neil C. R. Seiler M. W. Reynolds D. J. Smith J. J. Vaillancourt F. H. Smith P. G. (2022). Reprogramming RNA processing: An emerging therapeutic landscape. Trends Pharmacol. Sci. 43 (5), 437–454. 10.1016/j.tips.2022.02.011 35331569

[B75] O'Leary V. B. Ovsepian S. V. Smida J. Atkinson M. J. (2019). PARTICLE - The RNA podium for genomic silencers. J. Cell. Physiol. 234 (11), 19464–19470. 10.1002/jcp.28739 31058319

[B76] O'Leary V. B. Smida J. Buske F. A. Carrascosa L. G. Azimzadeh O. Maugg D. (2017). PARTICLE triplexes cluster in the tumor suppressor WWOX and may extend throughout the human genome. Sci. Rep. 7 (1), 7163. 10.1038/s41598-017-07295-5 28769061 PMC5541130

[B77] Oussalah A. Siblini Y. Hergalant S. Chéry C. Rouyer P. Cavicchi C. (2022). Epimutations in both the TESK2 and MMACHC promoters in the Epi-cblC inherited disorder of intracellular metabolism of vitamin B12. Clin. Epigenetics 14 (1), 52. 10.1186/s13148-022-01271-1 35440018 PMC9020039

[B78] Padmakumar S. Jones G. Khorkova O. Hsiao J. Kim J. Bleier B. S. (2021). Osmotic core-shell polymeric implant for sustained BDNF AntagoNAT delivery in CNS using minimally invasive nasal depot (MIND) approach. Biomaterials 276, 120989. 10.1016/j.biomaterials.2021.120989 34252799 PMC8607190

[B79] Park K. Zhong J. Jang J. S. Kim J. Kim H. J. Lee J. H. (2022). ZWC complex-mediated SPT5 phosphorylation suppresses divergent antisense RNA transcription at active gene promoters. Nucleic Acids Res. 50 (7), 3835–3851. 10.1093/nar/gkac193 35325203 PMC9023261

[B80] Partridge W. Xia S. Kwoh T. J. Bhanot S. Geary R. S. Baker B. F. (2021). Improvements in the tolerability profile of 2'-O-methoxyethyl chimeric antisense oligonucleotides in parallel with advances in design, screening, and other methods. Nucleic Acid. Ther. 31 (6), 417–426. 10.1089/nat.2020.0917 34242101 PMC8713270

[B81] Pisignano G. Ladomery M. (2021). Epigenetic regulation of alternative splicing: How LncRNAs tailor the message. Noncoding. RNA 7 (1), 21. 10.3390/ncrna7010021 33799493 PMC8005942

[B82] Podbevšek P. Fasolo F. Bon C. Cimatti L. Reißer S. Carninci P. (2018). Structural determinants of the SINE B2 element embedded in the long non-coding RNA activator of translation AS Uchl1. Sci. Rep. 8 (1), 3189. 10.1038/s41598-017-14908-6 29453387 PMC5816658

[B83] Przanowska R. K. Weidmann C. A. Saha S. Cichewicz M. A. Jensen K. N. Przanowski P. (2022). Distinct MUNC lncRNA structural domains regulate transcription of different promyogenic factors. Cell Rep. 38 (7), 110361. 10.1016/j.celrep.2022.110361 35172143 PMC8937029

[B84] Pu J. Zhang Y. Wang A. Qin Z. Zhuo C. Li W. (2021). ADORA2A-AS1 restricts hepatocellular carcinoma progression via binding HuR and repressing FSCN1/AKT Axis. Front. Oncol. 11, 754835. 10.3389/fonc.2021.754835 34733789 PMC8558402

[B85] Rinn J. L. Chang H. Y. (2020). Long noncoding RNAs: Molecular modalities to organismal functions. Annu. Rev. Biochem. 89, 283–308. 10.1146/annurev-biochem-062917-012708 32569523

[B86] Roberts T. C. Langer R. Wood M. J. A. (2020). Advances in oligonucleotide drug delivery. Nat. Rev. Drug Discov. 19 (10), 673–694. 10.1038/s41573-020-0075-7 32782413 PMC7419031

[B87] Romero-Barrios N. Legascue M. F. Benhamed M. Ariel F. Crespi M. (2018). Splicing regulation by long noncoding RNAs. Nucleic Acids Res. 46 (5), 2169–2184. 10.1093/nar/gky095 29425321 PMC5861421

[B88] Rowley M. J. Corces V. G. (2018). Organizational principles of 3D genome architecture. Nat. Rev. Genet. 19 (12), 789–800. 10.1038/s41576-018-0060-8 30367165 PMC6312108

[B89] Rydzik A. M. Gottschling D. Simon E. Skronska-Wasek W. Rippmann J. F. Riether D. (2021). Epigenetic modification 6-methyladenosine can impact the potency and specificity of siRNA. Chembiochem. 22 (3), 491–495. 10.1002/cbic.202000551 32936508

[B90] Sakamoto R. Jiang S. Tsukada Y. Tsujimoto H. Kimura T. (2019). IFN-Alpha1 antisense RNA represses human influenza A virus growth in a Guinea pig system. Front. Biosci. 24 (4), 798–818. 10.2741/4752 30844714

[B91] Seila A. C. Calabrese J. M. Levine S. S. Yeo G. W. Rahl P. B. Flynn R. A. (2008). Divergent transcription from active promoters. Science 322 (5909), 1849–1851. 10.1126/science.1162253 19056940 PMC2692996

[B92] Sergeeva O. V. Shcherbinina E. Y. Shomron N. Zatsepin T. S. (2022). Modulation of RNA splicing by oligonucleotides: Mechanisms of action and therapeutic implications. Nucleic Acid. Ther. 32 (3), 123–138. 10.1089/nat.2021.0067 35166605

[B93] Servais L. Mercuri E. Straub V. Guglieri M. Seferian A. M. Scoto M. and SKIP-NMD Study Group (2022). Long-term safety and efficacy data of golodirsen in ambulatory patients with Duchenne muscular dystrophy amenable to exon 53 skipping: A first-in-human, multicenter, two-part, open-label, phase 1/2 trial. Nucleic Acid. Ther. 32 (1), 29–39. 10.1089/nat.2021.0043 34788571 PMC8817703

[B94] Setten R. L. Chomchan P. Epps E. W. Burnett J. C. Rossi J. J. (2021). CRED9: A differentially expressed elncRNA regulates expression of transcription factor CEBPA. RNA 27 (8), 891–906. 10.1261/rna.078752.121 PMC8284328 34039742

[B95] Simone R. Javad F. Emmett W. Wilkins O. G. Almeida F. L. Barahona-Torres N. (2021). MIR-NATs repress MAPT translation and aid proteostasis in neurodegeneration. Nature 594 (7861), 117–123. 10.1038/s41586-021-03556-6 34012113 PMC7610982

[B96] Song H. Wang J. Wang X. Yuan B. Li D. Hu A. (2022). HNF4A-AS1-encoded small peptide promotes self-renewal and aggressiveness of neuroblastoma stem cells via eEF1A1-repressed SMAD4 transactivation. Oncogene 41 (17), 2505–2519. 10.1038/s41388-022-02271-4 35318442

[B97] Spencer E. R. Shi W. Komorowski R. W. Gilbert J. P. Ostrowski L. M. Bird L. M. (2022). Longitudinal EEG model detects antisense oligonucleotide treatment effect and increased UBE3A in Angelman syndrome. Brain Commun. 4 (3), fcac106. 10.1093/braincomms/fcac106 35611307 PMC9123847

[B98] Stolte B. Nonnemacher M. Kizina K. Bolz S. Totzeck A. Thimm A. (2021). Nusinersen treatment in adult patients with spinal muscular atrophy: A safety analysis of laboratory parameters. J. Neurol. 268 (12), 4667–4679. 10.1007/s00415-021-10569-8 33899154 PMC8563549

[B99] Su L. Shi Y. Y. Liu Z. Y. Gao S. J. (2022). Acute myeloid leukemia with CEBPA mutations: Current progress and future directions. Front. Oncol. 12, 806137. 10.3389/fonc.2022.806137 35178345 PMC8844020

[B100] Su Z. Liu G. Zhang B. Lin Z. Huang D. (2021). Natural antisense transcript PEBP1P3 regulates the RNA expression, DNA methylation and histone modification of CD45 gene. Genes (Basel) 12 (5), 759. 10.3390/genes12050759 34067766 PMC8156488

[B101] Suzuki S. Yuan H. Hirata-Tsuchiya S. Yoshida K. Sato A. Nemoto E. (2021). DMP-1 promoter-associated antisense strand non-coding RNA, panRNA-DMP-1, physically associates with EGFR to repress EGF-induced squamous cell carcinoma migration. Mol. Cell. Biochem. 476 (4), 1673–1690. 10.1007/s11010-020-04046-5 33420898

[B102] Tan C. P. Sinigaglia L. Gomez V. Nicholls J. Habib N. A. (2021). RNA activation-A novel approach to therapeutically upregulate gene transcription. Molecules 26 (21), 6530. 10.3390/molecules26216530 34770939 PMC8586927

[B103] Toki N. Takahashi H. Sharma H. Valentine M. N. Z. Rahman F. M. Zucchelli S. (2020a). SINEUP long non-coding RNA acts via PTBP1 and HNRNPK to promote translational initiation assemblies. Nucleic Acids Res. 48 (20), 11626–11644. 10.1093/nar/gkaa814 33130894 PMC7672464

[B104] Toki N. Takahashi H. Zucchelli S. Gustincich S. Carninci P. (2020b). Synthetic *in vitro* transcribed lncRNAs (SINEUPs) with chemical modifications enhance target mRNA translation. FEBS Lett. 594 (24), 4357–4369. 10.1002/1873-3468.13928 33012004

[B105] Vaasjo L. O. (2022). LncRNAs and chromatin modifications pattern m6A methylation at the untranslated regions of mRNAs. Front. Genet. 13, 866772. 10.3389/fgene.2022.866772 35368653 PMC8968631

[B106] Valentini P. Pierattini B. Zacco E. Mangoni D. Espinoza S. Webster N. A. (2022). Towards SINEUP-based therapeutics: Design of an *in vitro* synthesized SINEUP RNA. Mol. Ther. Nucleic Acids 27, 1092–1102. 10.1016/j.omtn.2022.01.021 35228902 PMC8857549

[B107] Vujovic F. Rezaei-Lotfi S. Hunter N. Farahani R. M. (2021). The fate of notch-1 transcript is linked to cell cycle dynamics by activity of a natural antisense transcript. Nucleic Acids Res. 49 (18), 10419–10430. 10.1093/nar/gkab800 34520549 PMC8501981

[B108] Wahlestedt C. (2013). Erratum: Targeting long non-coding RNA to therapeutically upregulate gene expression. Nat. Rev. Drug Discov. 12 (6), 433–446. Erratum in: Nat Rev Drug Discov. 2014;13(1):79. 10.1038/nrd4203 23722346

[B109] Wan L. Li W. Meng Y. Hou Y. Chen M. Xu B. (2022). Inflammatory immune-associated eRNA: Mechanisms, functions and therapeutic prospects. Front. Immunol. 13, 849451. 10.3389/fimmu.2022.849451 35514959 PMC9063412

[B110] Wang C. L. Li J. C. Zhou C. X. Ma C. N. Wang D. F. Wo L. L. (2021). Long non-coding RNA ARHGAP5-AS1 inhibits migration of breast cancer cell via stabilizing SMAD7 protein. Breast Cancer Res. Treat. 189 (3), 607–619. 10.1007/s10549-021-06286-5 34370213 PMC8505316

[B111] Wang H. Yang B. Cai X. Cheng X. Shen N. Liu L. (2021). Hepatocellular carcinoma risk variant modulates lncRNA HLA-DQB1-AS1 expression via a long-range enhancer-promoter interaction. Carcinogenesis 42 (11), 1347–1356. 10.1093/carcin/bgab095 34665859

[B112] Wang J. Bai J. OuYang S. Wang H. Jin Y. Peng X. (2021a). Antisense oligonucleotides targeting the SMN2 promoter region enhance SMN2 expression in spinal muscular atrophy cell lines and mouse model. Hum. Mol. Genet. 31, 1635–1650. ddab350. 10.1093/hmg/ddab350 34888619

[B113] Wang J. Cai Y. Lu H. Zhang F. Zheng J. (2021b). LncRNA APOA1-AS facilitates proliferation and migration and represses apoptosis of VSMCs through TAF15-mediated SMAD3 mRNA stabilization. Cell Cycle 20 (17), 1642–1652. 10.1080/15384101.2021.1951940 34382908 PMC8489913

[B114] Wang J. Yang X. Li R. Wang L. Gu Y. Zhao Y. (2018). Long non-coding RNA MYU promotes prostate cancer proliferation by mediating the miR-184/c-Myc axis. Oncol. Rep. 40 (5), 2814–2825. 10.3892/or.2018.6661 30132573

[B115] Wang T. Kong S. Tao M. Ju S. (2020). The potential role of RNA N6-methyladenosine in Cancer progression. Mol. Cancer 19 (1), 88. 10.1186/s12943-020-01204-7 32398132 PMC7216508

[B116] Wang W. Min L. Qiu X. Wu X. Liu C. Ma J. (2021a). Biological function of long non-coding RNA (LncRNA) xist. Front. Cell Dev. Biol. 9, 645647. 10.3389/fcell.2021.645647 34178980 PMC8222981

[B117] Wang W. Zhao Z. Xu C. Li C. Ding C. Chen J. (2021b). LncRNA FAM83A-AS1 promotes lung adenocarcinoma progression by enhancing the pre-mRNA stability of FAM83A. Thorac. Cancer 12 (10), 1495–1502. 10.1111/1759-7714.13928 33687144 PMC8107032

[B118] Wang X. Zhang M. Jiang L. Fang X. Zhang T. (2022). Exosomal AFAP1-AS1 binds to microRNA-15a-5p to promote the proliferation, migration, and invasion of ectopic endometrial stromal cells in endometriosis. Reprod. Biol. Endocrinol. 20 (1), 77. 10.1186/s12958-022-00942-1 35513844 PMC9069797

[B119] Wang X. Y. Yuan L. Li Y. L. Gan S. J. Ren L. Zhang F. (2018). RNA activation technique and its applications in cancer research. Am. J. Cancer Res. 8 (4), 584–593. 29736305 PMC5934550

[B120] Wang Y. Chen Z. (2022). Long noncoding RNA UBA6-AS1 inhibits the malignancy of ovarian cancer cells via suppressing the decay of UBA6 mRNA. Bioengineered 13 (1), 178–189. 10.1080/21655979.2021.2011640 34951345 PMC8805991

[B121] Wang Y. Mao Y. Zhao Y. Yi X. Ding G. Yu C. (2021). Early-life undernutrition induces enhancer RNA remodeling in mice liver. Epigenetics Chromatin 14 (1), 18. 10.1186/s13072-021-00392-w 33789751 PMC8011416

[B122] Wataya T. Takasaki S. Hoshino M. Makioka H. Nakamura G. Matsuda N. (2021). Real-world safety of nusinersen in Japan: Results from an interim analysis of a post-marketing surveillance and safety database. Int. J. Neurosci., 1–13. 10.1080/00207454.2021.1995382 34809526

[B123] Wei W. Zhao Q. Wang Z. Liau W. S. Basic D. Ren H. (2022). ADRAM is an experience-dependent long noncoding RNA that drives fear extinction through a direct interaction with the chaperone protein 14-3-3. Cell Rep. 38 (12), 110546. 10.1016/j.celrep.2022.110546 35320727 PMC9015815

[B124] Wijaya Y. O. S. Niba E. T. E. Nishio H. Okamoto K. Awano H. Saito T. (2022). High concentration or combined treatment of antisense oligonucleotides for spinal muscular atrophy perturbed SMN2 splicing in patient fibroblasts. Genes 13 (4), 685. 10.3390/genes13040685 35456491 PMC9027857

[B125] Xiao S. Huang Q. Ren H. Yang M. (2021). The mechanism and function of super enhancer RNA. Genesis 59 (5-6), e23422. 10.1002/dvg.23422 34028961

[B126] Xie W. Wang Y. Zhang Y. Xiang Y. Wu N. Wu L. (2021). Single-nucleotide polymorphism rs4142441 and MYC co-modulated long non-coding RNA OSER1-AS1 suppresses non-small cell lung cancer by sequestering ELAVL1. Cancer Sci. 112 (6), 2272–2286. 10.1111/cas.14713 33113263 PMC8177763

[B127] Xie X. Lin J. Fan X. Zhong Y. Chen Y. Liu K. (2021). LncRNA CDKN2B-AS1 stabilized by IGF2BP3 drives the malignancy of renal clear cell carcinoma through epigenetically activating NUF2 transcription. Cell Death Dis. 12 (2), 201. 10.1038/s41419-021-03489-y 33608495 PMC7895987

[B128] Xu W. He C. Kaye E. G. Li J. Mu M. Nelson G. M. (2022). Dynamic control of chromatin-associated m6A methylation regulates nascent RNA synthesis. Mol. Cell 82 (6), 1156–1168.e7. e7. 10.1016/j.molcel.2022.02.006 35219383 PMC8969783

[B129] Xue W. Wang F. Han P. Liu Y. Zhang B. Gu X. (2020). The oncogenic role of LncRNA FAM83C-AS1 in colorectal cancer development by epigenetically inhibits SEMA3F via stabilizing EZH2. Aging (Albany NY) 12 (20), 20396–20412. 10.18632/aging.103835 33109776 PMC7655168

[B130] Yang F. (2022). Promoter antisense RNAs: Beyond transcription by-products of active promoters. RNA Biol. 19 (1), 533–540. 10.1080/15476286.2022.2062177 35427206 PMC9037429

[B131] Yang F. Tanasa B. Micheletti R. Ohgi K. A. Aggarwal A. K. Rosenfeld M. G. (2021). Shape of promoter antisense RNAs regulates ligand-induced transcription activation. Nature 595 (7867), 444–449. 10.1038/s41586-021-03589-x 34194047 PMC8439151

[B132] Yang L. Chen Y. Liu N. Shi Q. Han X. Gan W. (2021). Low expression of TRAF3IP2-AS1 promotes progression of NONO-TFE3 translocation renal cell carcinoma by stimulating N^6^-methyladenosine of PARP1 mRNA and downregulating PTEN. J. Hematol. Oncol. 14 (1), 46. Erratum in: J Hematol Oncol. 2021;14(1):144. 10.1186/s13045-021-01059-5 33741027 PMC7980631

[B133] Yang W. Zhang K. Li L. Xu Y. Ma K. Xie H. (2021). Downregulation of lncRNA ZNF582-AS1 due to DNA hypermethylation promotes clear cell renal cell carcinoma growth and metastasis by regulating the N(6)-methyladenosine modification of MT-RNR1. J. Exp. Clin. Cancer Res. 40 (1), 92. Erratum in: J Exp Clin Cancer Res. 2021;40(1):167. 10.1186/s13046-021-01889-8 33691743 PMC7945252

[B134] Yang Y. Liu S. He C. Lv T. Zeng L. Zhang F. (2022). LncRNA LYPLAL1-AS1 rejuvenates human adipose-derived mesenchymal stem cell senescence via transcriptional MIRLET7B inactivation. Cell Biosci. 12 (1), 45. 10.1186/s13578-022-00782-x 35449031 PMC9022335

[B135] Ye J. Fu Y. Wang Z. Yu J. (2021). Long non-coding RNA FOXP4-AS1 facilitates the biological functions of hepatocellular carcinoma cells via downregulating ZC3H12D by mediating H3K27me3 through recruitment of EZH2. Cell Biol. Toxicol. 10.1007/s10565-021-09642-9 PMC9750913 34545456

[B136] Young R. S. Kumar Y. Bickmore W. A. Taylor M. S. (2017). Bidirectional transcription initiation marks accessible chromatin and is not specific to enhancers. Genome Biol. 18 (1), 242. 10.1186/s13059-017-1379-8 29284524 PMC5747114

[B137] Zarantonello G. Arnoldi M. Filosi M. Tebaldi T. Spirito G. Barbieri A. (2021). Natural SINEUP RNAs in autism spectrum disorders: RAB11B-AS1 dysregulation in a neuronal CHD8 suppression model leads to RAB11B protein increase. Front. Genet. 12, 745229. 10.3389/fgene.2021.745229 34880900 PMC8647803

[B138] Zhang B. Zhang M. Yang Y. Li Q. Yu J. Zhu S. (2022). Targeting KDM4A-AS1 represses AR/AR-Vs deubiquitination and enhances enzalutamide response in CRPC. Oncogene 41 (3), 387–399. 10.1038/s41388-021-02103-x 34759344 PMC8755543

[B139] Zhang H. Guan R. Zhang Z. Li D. Xu J. Gong Y. (2021). LncRNA nqo1-AS1 attenuates cigarette smoke-induced oxidative stress by upregulating its natural antisense transcript Nqo1. Front. Pharmacol. 12, 729062. 10.3389/fphar.2021.729062 34566651 PMC8456124

[B140] Zhang S. Zhao B. S. Zhou A. Lin K. Zheng S. Lu Z. (2017). m6A demethylase ALKBH5 maintains tumorigenicity of glioblastoma stem-like cells by sustaining FOXM1 expression and cell proliferation program. Cancer Cell 31 (4), 591–606. e6. 10.1016/j.ccell.2017.02.013 28344040 PMC5427719

[B141] Zhao H. Xu Q. (2020). Long non-coding RNA DLX6-AS1 mediates proliferation, invasion and apoptosis of endometrial cancer cells by recruiting p300/E2F1 in DLX6 promoter region. J. Cell. Mol. Med. 24 (21), 12572–12584. 10.1111/jcmm.15810 32951317 PMC7686961

[B142] Zhao Y. Riching A. S. Knight W. E. Chi C. Broadwell L. J. Du Y. (2022). Cardiomyocyte-specific long noncoding RNA regulates alternative splicing of the triadin gene in the heart. Circulation 146, 699–714. 10.1161/CIRCULATIONAHA.121.058017 35862102 PMC9427731

[B143] Zheng F. Chen J. Zhang X. Wang Z. Chen J. Lin X. (2021). The HIF-1α antisense long non-coding RNA drives a positive feedback loop of HIF-1α mediated transactivation and glycolysis. Nat. Commun. 12 (1), 1341. 10.1038/s41467-021-21535-3 33637716 PMC7910558

[B144] Zhou N. Li S. Wu D. Zhang F. Tang F. Li Y. (2021). The lncRNA VPS9D1-AS1 promotes hepatocellular carcinoma cell cycle progression by regulating the HuR/CDK4 Axis. DNA Cell Biol. 40 (10), 1278–1289. 10.1089/dna.2021.0235 34558987

[B145] Zhou Y. Xu S. Zhang M. Wu Q. (2021). Systematic functional characterization of antisense eRNA of protocadherin α composite enhancer. Genes Dev. 35 (19-20), 1383–1394. 10.1101/gad.348621.121 34531317 PMC8494205

[B146] Zong X. Nakagawa S. Freier S. M. Fei J. Ha T. Prasanth S. G. (2016). Natural antisense RNA promotes 3' end processing and maturation of MALAT1 lncRNA. Nucleic Acids Res. 44 (6), 2898–2908. 10.1093/nar/gkw047 26826711 PMC4824109

